# Hexameric assembly of the AAA+ protein McrB is necessary for GTPase activity

**DOI:** 10.1093/nar/gky1170

**Published:** 2018-12-06

**Authors:** Neha Nirwan, Pratima Singh, Gyana Gourab Mishra, Christopher M Johnson, Mark D Szczelkun, Katsuaki Inoue, Kutti R Vinothkumar, Kayarat Saikrishnan

**Affiliations:** 1 Division of Biology, Indian Institute of Science Education and Research, Pune 411008, India; 2 MRC Laboratory of Molecular Biology, Cambridge CB2 0QH, UK; 3 DNA-Protein Interactions Unit, School of Biochemistry, Medical Sciences Building, University of Bristol, Bristol BS8 1TD, UK; 4 Diamond Light Source, Harwell Science and Innovation Campus, Fermi Avenue, Didcot OX11 0DE, UK

## Abstract

McrBC is one of the three modification-dependent restriction enzymes encoded by the *Escherichia coli* K12 chromosome. Amongst restriction enzymes, McrBC and its close homologues are unique in employing the AAA+ domain for GTP hydrolysis-dependent activation of DNA cleavage. The GTPase activity of McrB is stimulated by the endonuclease subunit McrC. It had been reported previously that McrB and McrC subunits oligomerise together into a high molecular weight species. Here we conclusively demonstrate using size exclusion chromatography coupled multi-angle light scattering (SEC-MALS) and images obtained by electron cryomicroscopy that McrB exists as a hexamer in solution. Furthermore, based on SEC-MALS and SAXS analyses of McrBC and the structure of McrB, we propose that McrBC is a complex of two McrB hexamers bridged by two subunits of McrC, and that the complete assembly of this complex is integral to its enzymatic activity. We show that the nucleotide-dependent oligomerisation of McrB precedes GTP hydrolysis. Mutational studies show that, unlike other AAA+ proteins, the catalytic Walker B aspartate is required for oligomerisation.

## INTRODUCTION

Prokaryotes employ different strategies to protect themselves against bacteriophage attack. One of these defence systems are the Modification Dependent Restriction (MDR) enzymes (1,2). Unlike the more widespread and well-studied restriction-modification (RM) systems, MDR enzymes cleave DNA containing target sequences with a modified (e.g. methylated) base. Interestingly, the description of host-induced non-hereditary variation in bacteriophages by Salvador Luria and Mary Human in 1952 (3), which eventually led to the discovery of the phenomenon of DNA restriction, was actually due to the action of an MDR enzyme. It was later shown that the observations made by Luria and Human were due to the activity of the enzyme McrBC (4–7) which is one of the three modification dependent RM enzyme systems encoded by *Escherichia coli* K12 chromosome (7), the other two being McrA and Mrr (2).

The *mcrbc* locus in the *E. coli* K12 chromosome has two overlapping genes, *mcrb* and *mcrc*, which code for the 54 kDa McrB protein and the 40 kDa McrC protein, respectively. The N-terminal 161 residues of McrB form the DNA binding domain, while the rest constitute a GTP binding AAA+ domain (8–12). The nucleolytic active site is located in McrC, which belongs to the PD-(D/E)xK family of nucleases (13). The two proteins in presence of GTP form the McrBC complex (11). McrBC recognises and cleaves DNA having 5-hydroxymethylcytosine, 5-methylcytosine or 4-methylcytosine preceded by a purine (R^m^C) (7,14), making it a useful tool for studying epigenetic modifications (15). DNA cleavage requires at least two recognition sites, which can be 40 bp to 3000 bp apart (14,16). The cleavage happens close to one of the sites and hydrolysis of GTP is necessary (13,17).

McrBC is unique among the restriction enzymes in employing a GTP hydrolysing AAA+ motor to catalyse endonucleolytic cleavage. It has been proposed that motor-driven translocation of double-stranded (ds) DNA by the AAA+ motor of a target bound McrBC culminates in DNA cleavage upon collision with another translocating McrBC (11,17). Translocation of substrates by AAA+ motors are essential for a number of biological processes such as proteolysis by ClpX, Lon, FtsH etc. (18); DNA unwinding activity of superfamily 3 (SF3) helicases and SF6 helicases (e.g. replicative helicase MCM) (19); bacterial chromosome segregation by FtsK; and viral capsid packaging (20). McrBC serves as a model system to understand the mechanism of NTPase activity in driving long-range communication along DNA and the coupled nucleolytic activity.

The McrB GTPase motor belongs to the clade VI of AAA+ proteins, characterised by a pre-sensor β-hairpin insertion and an additional β-hairpin inserted in helix 2 of the canonical core (18). In almost all of the diverse functions of AAA+ proteins, oligomerisation of the AAA+ domains is required for activity. For example, structural studies of the hexameric SF3 helicase E1 from papillomavirus in complex with a DNA substrate mimic revealed the essentiality of the oligomeric structure and cooperation between six subunits to translocate single-stranded DNA (21). Oligomerisation has also been observed for the endonucleolytic complex McrBC (11,13,14). In an earlier study, using size exclusion chromatography (SEC), negative stain electron microscopy (EM) and mass analysis by scanning transmission electron microscopy (STEM), it was proposed that McrB probably forms a heptameric ring-like structure in presence of GTP (11). Addition of McrC then resulted in the formation of a proposed tetradecameric McrBC oligomer made of two heptameric rings of McrB and two subunits of McrC (11). A variant of McrB that lacks the N-terminal DNA-binding domain, McrB_s_, was also reported to form a heptameric ring similar to the full-length protein (11), while isolated N-terminal domain was found to be monomeric (12) consistent with oligomerisation of the AAA+ domains. The relative specificity of the DNA-binding domain to various cytosine modifications was also recently reported (22).

In contrast, AAA+ proteins predominantly form a hexameric ring or a spiral/lockwasher structure, and hence the heptamer of McrB is unusual (18). Although, heptameric rings have been reported for other AAA+ proteins (23–29), their functional significance is under debate (29–35). In almost all the cases, the functional assembly has been shown to be hexameric (33). In AAA+ proteins, nucleotide binding and hydrolysis happens at the interface between two neighbouring protomers of the oligomer. The relative orientation of the protomers effects the proper placement of the catalytically important residues for nucleotide hydrolysis. The relative orientation of the protomers, and consequently the mechanism of hydrolysis, would be affected by whether the oligomeric assembly is a hexamer or a heptamer. Furthermore, the number of protomers will affect the geometry of the assembly, in particular the pore in the assembly, which is often a functionally important site (18).

As part of our efforts to understand the molecular mechanism of the restriction enzyme McrBC, we first sought to clarify the assembly of the McrB and McrBC oligomers. Here we report the results of purification, functional characterisation, and accurate measurement of the molecular masses of oligomeric McrB and McrBC. In combination with electron cryomicroscopy (cryoEM) studies, we show that McrB in presence of GTP forms a hexamer rather than a heptamer. We show that the GTPase activity of the enzyme is initiated upon nucleotide-dependent oligomeric assembly of McrBC. Based on the detailed study of the oligomeric assembly of McrBC, we propose a model for the assembly of McrBC complex in presence of GTP.

## MATERIALS AND METHODS

### Cloning of mcrB, mcrBΔN, mcrC

*mcrB* and *mcrC* were amplified by PCR from genomic DNA of *Escherichia coli* K12 using forward and reverse primers 5′- CTTTAAGAAGGAGATATACATATGGAATCTA TTCAACCCTGGATTG-3′ and 5′- GATGATGGGATCCCGATGAGTCCCC-3′, and 5′- CTTTAAGAAGGAGATATACATATGGAACAGCCCGTGATACC-3′ and 5′- GATGATGG GATCCTTATTTGAGATATTC-3′ respectively. The amplified products were cloned into pHIS17 vector (36) using a restriction-free cloning method (37). *mcrBΔN* was amplified from *mcrB* gene in pHIS17 vector by using forward and reverse primers-5′-GTTTAACTTTAAGAAGGAGATATACATATGTCAAAAACTGAATCATACTG-3′ and 5′-GATGATGGGATCCCGATGAGTCCCC-3′, respectively. *mcrBΔN* lacking the C-terminal histidine tag i.e. *mcrBΔN^WT^* (without tag) was amplified using forward and reverse primers, 5′-GTTTAACTTTAAGAAGGAGATATACATATGTCAAAAACTGAATCATACTG-3′ and 5′-TTAATGATGATGATGATGATGGGATCCCTATGAGTCCCCTAATAATTTGTTGG-3′, respectively, and cloned into pHIS17 vector using restriction-free cloning method (37). The resulting *mcrB-his_6_, mcrBΔN-his_6,_ mcrBΔN^WT^* and *mcrC* genes were fully sequenced.

All the mutations were performed using restriction-free cloning method. Supplementary Table S1 lists the sequence of the primers used to introduce the mutations. These PCR amplified fragments with the mutations were used as primers in a second PCR reaction and a plasmid containing McrB wild-type gene (pHISMcrB) was used as a template to obtain full-length mutant genes. These amplified products were used for restriction-free cloning. All the genes were sequenced to ensure only the desired mutations were incorporated.

### Purification of McrB, McrBΔN and McrC

McrB and McrBΔN were expressed with a tag of six histidines at C-terminus and McrC without tag by overexpression of pHISMcrB, pHISMcrBΔN and pHISMcrC plasmids, respectively, in *E. coli* BL21 (AI) cells (Invitrogen). The tag was preceded by a glycine and a serine. The cultures were grown in 2L LB media containing 100 μg/ml ampicillin in an incubator-shaker at 37°C until OD reached 0.3 at 600 nm. The temperature of incubator-shaker was then reduced to 18°C and cultures were induced with 0.06% w/v L-Arabinose. The cultures were grown further overnight (15–16 h) at 18°C. Cells were pelleted by centrifugation at 4°C and 3315 g for 15 min. The pellet was resuspended in 50 ml lysis buffer (50 mM Tris–Cl pH 8, 25 mM imidazole, 500 mM NaCl, 5 mM MgCl_2_, 10% (v/v) glycerol). For McrB and McrBΔN, 0.04% (w/v), CHAPS was added to the cell pellet resuspension. The cells were lysed by sonication at 4°C. The cell lysate was then clarified by ultracentrifugation at 4°C and 159 200 g for 40 min. Though McrC did not have a His-tag, while working toward a purification protocol, we found that the protein bound to NiNTA column (GE Life Sciences), which helped in the purification. Consequently, all the three proteins were first purified by NiNTA column using an identical strategy. The clarified supernatant of the cell lysate was loaded onto a 5 ml NiNTA column equilibrated with Buffer A (50 mM Tris–Cl pH 8, 25 mM imidazole, 500 mM NaCl). The protein was eluted using Buffer A and Buffer B (50 mM Tris–Cl pH 8, 500 mM Imidazole, 500 mM NaCl) by a step gradient from 5% to 100% at an interval of 20%. The purest of the NiNTA fractions, inferred from SDS-PAGE analysis, were dialysed against 2 l dialysis buffer (50 mM Tris–Cl pH 8, 50 mM NaCl, 1 mM EDTA and 1 mM DTT).

Dialysed McrB or McrBΔN were loaded onto an 8 ml MonoQ 10/100 GL column (GE Life Sciences) equilibrated with Buffer B50 (50 mM Tris–Cl pH 8, 50 mM NaCl, 1 mM EDTA, 1 mM DTT). 2 ml fractions were collected in 20 column volumes over a linear gradient of 0–50% of buffer B1000 (50 mM Tris–Cl pH 8, 1000 mM NaCl, 1 mM EDTA, 1 mM DTT) mixed with buffer B50. The pure fractions were pooled based on SDS-PAGE analysis and concentrated using a 2 ml 10 kDa vivaspin2 concentrator (GE Life Sciences). The concentrated protein was then incubated with 2.5 mM GTP, 5 mM MgCl_2_ in buffer B100^−GTP^ (50 mM Tris–Cl pH 8, 100 mM NaCl, 1 mM DTT) for 10 minutes at room temperature before loading onto 24 ml Superdex200 10/300 GL column (GE Life Sciences), equilibrated with buffer B100^+GTP^ (50 mM Tris–Cl pH 8, 100 mM NaCl, 0.1mM GTP, 5 mM MgCl_2_, 1 mM DTT). Pure fractions were pooled and concentrated using a 2 ml 10 kDa vivaspin2 concentrator. The concentrated protein was washed with storage buffer (100 mM NaCl, 10 mM Tris–Cl pH 7.4 and 1 mM DTT) to remove GTP. Washing was performed by adding 1.5 ml storage buffer to 0.5 ml concentrated protein. The diluted sample was then concentrated to 0.5 ml again using a 2 ml 10 kDa vivaspin2 concentrator (GE Life Sciences). The process was repeated 3–5 times to minimise GTP contamination. The purity of protein was assessed by calculating the ratio of UV absorption at 260 and 280 nm, which changed from 1.5 (GTP-containing sample) to 0.6–0.5 (washed sample). The proteins were stored in storage buffer at −80°C. McrBΔN without His_6_ tag was also purified (data not shown). The same protocol was used for purification of the mutants of McrB.

McrC was further purified using 8 ml MonoS 10/100 GL column (GE Life Sciences) equilibrated with Buffer B50. 2 ml fractions were collected in 20 column volumes over a gradient of 0% to 50% of buffer B1000 mixed with buffer B50. The pure fractions, inferred from SDS-PAGE analysis, were pooled and concentrated using a 2 ml 10 kDa vivaspin2 concentrator. The concentrated protein was washed with storage buffer (see above) to remove EDTA and was stored in storage buffer at −80°C.

### Purification of McrBΔN^WT^

McrBΔN^WT^ was overexpressed using pHISMcrBΔN plasmid in *E. coli* BL21 (AI) cells. The culture was grown in 2 l LB media containing 100 μg/ml ampicillin in an incubator-shaker at 37°C until OD reached 0.3 at 600 nm. The temperature of incubator-shaker was then reduced to 18°C and cultures were induced with 0.06% (w/v) l-arabinose. The cultures were grown further overnight (15–16 h) at 18°C. Cells were pelleted by centrifugation at 4°C and 3315 g for 15 min. The pellet was resuspended in 50 ml lysis buffer (50 mM Tris–Cl pH 8, 100 mM NaCl, 5 mM MgCl_2_, 10% glycerol, 0.04% CHAPS and 1 mM DTT). The cell lysate was then clarified by ultracentrifugation at 4°C and 159 200 g for 40 min. The clarified supernatant of the cell lysate was loaded onto three columns connected in series- 5 ml HiTrap™ Heparin column (GE Life Sciences), 5 ml HiTrap™ SP HP (GE Life Sciences), 5 ml HiTrap Q HP (GE Life Sciences). The columns were equilibrated with Buffer B50 (50 mM Tris–Cl pH 8, 50 mM NaCl, 1 mM EDTA, 1 mM DTT) before loading the supernatant. Flow through from this step was collected and 45% ammonium sulfate was added followed by centrifugation in SS34 tubes placed in JA 25.5 rotor (Avanti High-Speed centrifuge) at 21 000 g, 4°C for 20 min. The final ammonium sulphate concentration of the supernatant (45% ammonium sulphate) was made to 70% and again centrifuged in SS34 tubes placed in JA 25.5 rotor (Avanti High-Speed centrifuge) at 21 000 g, 4°C for 20 min. The pellet from 75% ammonium sulphate precipitation was resuspended in 500 ml Buffer B0 (50 mM Tris–Cl pH 8, 1 mM EDTA, 1 mM DTT) and loaded onto a 5 ml HiTrap DEAE FF (GE Life Sciences) column equilibrated with Buffer B50. 4 ml fractions were collected in 20 column volumes over a linear gradient of 0% to 100% of buffer B1000 (50 mM Tris–Cl pH 8, 1000 mM NaCl, 1 mM EDTA and 1 mM DTT). The fractions with the highest purity, based on SDS-PAGE analysis, were pooled and equal amount of buffer B50^+2M(NH4)2SO4^ (50 mM Tris–Cl pH 8, 50 mM NaCl, 1 mM EDTA, 1 mM DTT and 2 M ammonium sulphate) was added. The protein solution was then loaded onto a 5 ml HiTrap Phenyl FF (low substitution) column (GE Life Sciences) equilibrated with buffer B50^+2M(NH4)2SO4^. 2 ml fractions were collected in 20 column volumes over a linear gradient of 0% to 100% of buffer B50. Pure fractions were dialysed against 2 l dialysis buffer (50 mM Tris–Cl pH 8, 50 mM NaCl, 1 mM EDTA, and 1 mM DTT) overnight. Dialysed McrBΔN protein solution was loaded onto an 8 ml MonoQ 10/100 GL column (GE Life Sciences) equilibrated with Buffer B50. 2 ml fractions were collected over 20 column volumes using a linear gradient of 0% to 50% of buffer B1000. The pure fractions were pooled and concentrated using a 2 ml 10 kDa vivaspin2 concentrator (GE Life Sciences). Concentrated sample (500 μl) was washed with 5 ml buffer B100 (50 mM Tris–Cl pH 8, 100 mM NaCl and 1 mM DTT) to remove EDTA. The concentrated protein was then incubated with 2.5 mM GTP, 5 mM MgCl_2_ for 10 min at room temperature. Sample was centrifuged for 15 min at 12 000 g, 4°C before loading onto 24 ml Superdex200 10/300 GL column (GE Life Sciences) equilibrated with buffer B100^+GTP^. Pure fractions, decided based on SDS-PAGE analysis, were pooled and concentrated using a 2 ml 10 kDa Vivaspin2 concentrator (GE Life Sciences). The concentrated protein was washed with storage buffer (100 mM NaCl, 10 mM Tris–Cl pH 7.4 and 1 mM DTT) to remove GTP. The concentration of protein was estimated using Bradford reagent with BSA as standard (38). The purified protein was stored at −80°C.

### Purification of McrBC/McrBΔNC complex

After purifying the individual subunits, the complex of McrB or McrBΔN with McrC was assembled and purified through size exclusion chromatography. McrB or McrBΔN was mixed with McrC at 4 fold higher molar concentration (i.e 4:1 ratio) and incubated with 2.5 mM GTP and 5 mM MgCl_2_ in buffer B100^−GTP^ for 10 min at room temperature. Sample was centrifuged at 21 000 g for 15 min before loading onto 120 ml Superdex200 10/300 GL column (GE Life Sciences), equilibrated with buffer B100^+GTP^. Pure fractions, decided based on SDS-PAGE analysis, were pooled and concentrated using a 2 ml 10 kDa Vivaspin2 concentrator (GE Life Sciences). The concentrated protein was washed with storage buffer to remove GTP. Protein concentration was estimated using both Bradford reagent (38) with BSA as standard and absorption at 280 nm. Absorption at 260 nm was also measured to check for bound nucleotide contamination. The concentrated complex was stored in storage buffer at −80°C.

### Size exclusion chromatography

Analysis of the oligomeric status of McrB and McrBΔN was carried out by Size exclusion chromatography (SEC) using 24 ml Superdex 200 10/300 GL either in the presence or absence of GTP. For studies without nucleotide the column was equilibrated with buffer B100^−GTP^, and for studies with nucleotide the column was equilibrated with B100^+GTP^. For both McrB and McrBΔN, 500 μl solution containing 18 μM protein in buffer B100^−GTP^ was injected with or without 2.5 mM nucleotide GTP and 5 mM MgCl_2_. The column was calibrated using a set of standard protein solutions. Blue dextran 2000 was used to determine void volume (*V*_o_ = 8.4 ml). The standards used for calibration of molecular masses were β amylase (200 kDa; *V*_e_ = 12.3 ml), alcohol dehydrogenase (150 kDa; *V*_e_ = 13.3 ml), bovine serum albumin (66 kDa; *V*_e_ = 14.3 ml), ovalbumin (43 kDa; *V*_e_ = 15.7 ml) and carbonic anhydrase (29 kDa; *V*_e_ = 16.8 ml). The chromatographic partition coefficient for SEC, *K*_av_, was calculated as *K*_av_ = *V*_e_ – *V*_o_/*V*_t_ – *V*_o_, where *V*_t_ = 24 ml (total column volume). Molecular mass was determined by fitting straight line through the standard curve of *K*_av_ versus logarithm of molecular weight standards (*R*^2^ = 0.9867) (Supplementary Figure S1).

The oligomerisation of McrB in presence of McrC and GTP was studied using a 24 ml Superose 6 10/300 GL SEC column (GE Life Sciences). The column was equilibrated with B100^+GTP^. 500 μl solution containing 18 μM McrB or McrBΔN and 4.5 μM McrC (4:1 ratio) in B100^−GTP^ with 2.5 mM GTP and 5 mM MgCl_2_ was incubated for 10 min at room temperature before injection.

### SEC-MALS

The mass in solution of McrB, McrBΔN, McrBC and McrBΔNC was determined by SEC-Multi-Angle Light Scattering (MALS) measurements using a Wyatt Heleos II 18 angle light scattering instrument coupled to a Wyatt Optilab rEX online refractive index detector. Detector 12 in the Heleos instrument was replaced with Wyatt's QELS detector for dynamic light scattering measurement. Protein samples (100 μl) were resolved using a Superdex S-200 (McrB) or Superose 6 (McrBC) 10/300 analytical gel filtration column (GE Healthcare) running at 0.5 ml/min in 10 mM Tris–Cl pH 7.4, 100 mM NaCl, 1 mM MgCl_2_, 1 mM DTT and 0.1 mM GTP buffer before passing through the light scattering and refractive index detectors in a standard SEC-MALS format.

Protein concentration was determined from the excess differential refractive index (RI) based on 0.186 × 10^−3^ RI increment for 1 mg/ml protein solution. The concentration and the observed scattered intensity at each point in the chromatograms were used to calculate the absolute molecular mass from the intercept of the Debye plot using Zimm's model as implemented in Wyatt's ASTRA software. Autocorrelation analysis of data from the dynamic light scattering detector was also performed using Wyatt's ASTRA software and the translational diffusion coefficients determined were used to calculate the hydrodynamic radius (*R*_h_) using the Stokes-Einstein equation and the measured solvent viscosity of 9.3e^−3^ Poise.

### Small angle X-ray scattering measurements

All SAXS measurements were performed on beamline B21 at Diamond Light Source (Didcot, Oxfordshire, UK). The sample-to-detector distance was 4.0 m and X-ray wavelength was 1 Å. SAXS data was recorded with 2 dimensional detector (PILATUS 2M, Dectris) at 15°C. Prepared samples were put on a 96-well plate and 20 μl of sample was injected from each well to the exposure cell by the automated sample changer (BioSAXS robot). Measurements were taken for 1 mg/ml protein sample in buffer B100. Each scattering curve was an average of 18 frames (10 s exposure/frame). Data were processed and analysed using SCATTER (39).

### Electron cryomicroscopy

Full length and the N-terminally truncated McrB oligomers were assembled by addition of 1 mM GDPNP and 1 mM MgCl_2_ at a final concentration of 2.5 mg/ml in 10 mM Tris–Cl pH 7.4 and 0.1 M NaCl. 3 μl of the assembled complex was applied to a glow discharged Quantifoil grids R 0.6/1 or 1.2/1.3 and blotted for 11 s, then plunge-frozen in liquid ethane using an environmental plunge-freeze apparatus (40). McrBΔN were transferred to Krios cartridges and imaged with a FEI Titan Krios electron microscope and Falcon II direct detector at 300 keV with the specimen temperature at −186°C at a calibrated magnification of 105 263× (nominal magnification is 59 000), corresponding to a 1.33 Å/pixel with a 2.5 s exposure. The full-length McrB was imaged on the Polara microscope at 300 kEV equipped also with a Falcon II detector with the specimen temperature at −186°C at a calibrated magnification of 104 477× (nominal magnification is 78 000), corresponding to a 1.34 Å/pixel with a 3 s exposure.

Initial processing was done with EMAN2 (41). Particles from both data sets were picked with e2boxer and extracted with a box size of 160 pixels. Preferred orientation was observed and only top/bottom views were picked. A total of 1568 and 3350 particles from the full length and truncated McrB were subjected to reference-free 2D class averaging. Subsequent processing with a larger data set was done with RELION 2.1 (42). The CTF of the images were estimated with GCTF (43) and the particles were automatically picked with Gautomatch (http://www.mrc-lmb.cam.ac.uk/kzhang/) using the 2D class averages from EMAN2 as templates. A total of 14 498 and 10 417 particles of McrBdN and McrB respectively were picked and extracted with a box size of 160 pixels. These were subjected to reference-free 2D class averaging with the resolution during refinement limited to 15 Å to prevent overfitting. Bad particles were removed and the final data set had 9478 and 9774 particles for McrBdN and McrB, respectively. The oligomeric state of both versions of McrB in class averages was further checked with rfiltim (44,45). Due to preferred orientation of the particles further processing were not performed.

### GTPase assay

The hydrolysis of GTP was qualitatively measured by monitoring release of phosphate ions (P_i_) using a standard malachite green assay (46,47). Each GTPase assay was performed in triplicate. A master mix containing protein and 1 mM GTP (Jena bioscience) in hydrolysis buffer (10 mM Tris–Cl pH 8, 50 mM KCl, 15 mM MgCl_2_, 1 mM DTT) was incubated at 37°C. To check the effect of DNA on the GTPase activity, a final concentration of 1 μM of either the 60 bp specific or the 62 bp non-specific DNA was added (see below for sequence details). 20 μl volume of the reaction mix was withdrawn at regular time intervals and 5 μl of 0.5 M EDTA was added to stop the reaction at each time point. The samples were then transferred to a 96 well flat bottom plate. 50 μl of freshly prepared malachite green mix (800 μl malachite green solution, 200 μl of 7.5% (w/v) ammonium molybdate and 16 μl of 11% (v/v) Tween 20) was added to each reaction and incubated for 10 min at room temperature. Absorbance was measured at 630 nm in Varioscan plate reader.

Malachite green solution was prepared by adding 44 mg malachite green carbinol base (Sigma Aldrich) powder to 36 ml 3N sulphuric acid solution. A reaction mixture quenched with EDTA at 0 min was used as a blank and was included for every set of reactions. This blank reading was comparable to the absorbance measured for hydrolysis buffer containing 1 mM GTP. Blank absorbance reading was taken at the end of one hour and subtracted from all the absorbance readings to rule out spontaneous GTP hydrolysis at 37°C. To measure the amount of Pi released (μM), standard phosphate curve was plotted by preparing different dilutions of a 2 M aqueous NaH_2_PO_4_ solution. 50 μl of malachite green solution was added to 25 μl of each of the dilutions and the reaction was incubated for 10 minutes at room temperature (Supplementary Figure S2A).

### DNA cleavage assay

A 114 bp substrate MB114MspI was generated by overlap PCR using MB60MSPI-1F (5′-GCCGGGTAACCGGGTAAGTCCGGGTAAGA^m^CCGGTAGTTCGGATCGAGGGGT AGGCCGC-3′) and MB60MSPI-2R (5′-AGTCAAATTGCATATGCTGGTCTTTCAGCG^m^CCGGTAATCGTCTTGTGAAGGATCCGCGGC-3′) as primers. The duplex MB114MspI was purified by gel extraction from a 2% (w/v) agarose gel. Nucleolytic cleavage of DNA was carried out in 10 μl reaction mix of a digestion buffer (10 mM Tris–Cl pH 8, 50 mM KCl, 15 mM MgCl_2_, 1 mM DTT) containing 75 nM MB114MspI incubated with protein in presence or absence of 1 mM GTP (Jena bioscience). The reactions were incubated at 37°C for 30 min. 2 μl 6X STES buffer (40% (w/v) sucrose, 0.2 M Tris–Cl pH 7.5, 40 mM EDTA, 1% (w/v) SDS) was added before loading on a native 10% (w/v) polyacrylamide gel (pre-electrophoresed for 30 minutes at room temperature in 1XTBE buffer). The gel was run at 230 V for 40 min, and then stained with a solution containing 2 μg/ml ethidium bromide for 5 min and imaged on Typhoon TRIO+ variable mode imager at high sensitivity.

### Stopped-flow pre-steady-state GTPase rate measurements

GTP hydrolysis by McrB and McrB+McrC were measured by using the phosphate binding protein (PBP) labeled with N-[2-(1-maleimidyl)ethyl]-7-(diethylamino)coumarin-3-carboxamide (MDCC) (48). PBP was expressed, purified and labelled as described previously (49). The fluorescent N-[2-(1-maleimidyl)ethyl]-7-(diethylamino)coumarin-3-carboxamide (MDCC) label was obtained from Sigma. The labeled PBP shows an increase in fluorescence upon binding to phosphate. A calibration curve was prepared with an inorganic phosphate standard and experimental data were analysed within the linear range of the standard (Supplementary Figure S3). Inorganic phosphate contamination in the nucleotide was removed by treatment with a phosphate ‘mop’ (50) – bacterial purine nucleoside phosphorylase (PNPase) and 7-methylguanosine (7-MG) (Sigma) for 1 h followed by filtering the mopped solution using a protein concentrator (Vivaspin20, 10 kDa Sartorius). Fluorescence intensity was measured using SF61-DX2 stopped-flow fluorimeter (TgK Scientific, UK) with excitation at 436 nm (2 nm bandwidth) and a 455 nm long-pass filter (Schott GG455). All measurements were performed at 25°C. In each reaction, equal volume of 12 μM PBP-MDCC from syringe C and protein from syringe D were mixed, making the final concentration of PBP-MDCC as 6 μM. All GTPase experiments were carried out in buffer containing 50 mM KCl, 10 mM Tris–Cl pH 8, 5 mM MgCl_2_ and 1 mM DTT. Equal volume of reactants was mixed from both syringe C and D. The concentration of nucleotide and protein post mixing is mentioned with each data in the text.

### Steady-state tryptophan fluorescence measurements

Steady-state tryptophan fluorescence was measured at 25°C using Horiba FluoroMax 4 spectrometer with λ_ex_ = 297 nm (slit width 5 nm). Emission spectra was collected from 307 to 407 nm (slit width = 5 nm). Final concentration of reactants (as mentioned in different experiments) were 500 nM McrB, 125 nM McrC, 1 mM nucleotide (GTP, GDP, GDPNP or GTPγS) and 500 nM mDNA in 50 mM KCl, 10 mM Tris pH 8, 5 mM MgCl_2_ and 1 mM DTT.

### Correcting inner filter effect

The inner filter effect of different ligands (nucleotides or DNA) was corrected by using 500 nM tryptophan solution with 1 mM nucleotide (GTP, GDP, GDPNP or GTPγS) or 500 nM DNA (specific mDNA1) in 50 mM KCl, 10 mM Tris–Cl pH 8, 5 mM MgCl_2_ and 1 mM DTT.

### Stopped-flow intrinsic tryptophan fluorescence kinetic study

Tryptophan fluorescence was measured at 25°C using the SF61-DX2 stopped-flow fluorimeter (TgK Scientific, UK) with excitation at 297 nm (4 nm bandwidth) and a 320 nm long-pass filter (Schott WG320). Reactants in all experiments were mixed in 1:1 ratio from syringe C and syringe D and the reaction was monitored over a time period of 0–180 s in logarithmic acquisition mode. Final concentrations of reactants in all experiments were 500 nM McrB/McrBΔN, 125 nM McrC, 1 mM GTP, in 50 mM KCl, 10 mM Tris–Cl pH 8, 5 mM MgCl_2_ and 1 mM DTT.

### Nucleotide binding assay using Mant-GDP

The fluorescent nucleotide analog 2′/3′-*O*-(*N*-methyl-anthraniloyl)-guanosine-5′-diphosphate (Mant-GDP) (51) was obtained from Jena biosciences. Fluorescence emission spectra were recorded on Horiba FluoroMax^®^ 4 spectrophotometer (Jobin Yvon) at 25°C in a 10 × 10 mm quartz cuvette. The excitation wavelength was set at 360 nm and single point intensities were measured at 440 nm (*I*_440_). For the fluorescence measurements, 0.5 μM protein in buffer (50 mM KCl, 10 mM Tris–Cl pH 8, 5 mM MgCl_2_ and 1 mM DTT) was added to the cuvette and a blank spectrum was taken. Mant-GDP was added to the protein gradually with increasing concentration steps and *I*_440_ were recorded for each concentration. Before each measurement, the protein was incubated with Mant-GDP for one minute prior to collection of the spectra. *I*_440_ readings of Mant-GDP without protein were taken as control. The difference of *I*_440_ in presence and absence of protein were plotted against Mant-GDP concentration.

### Circular-dichroism spectroscopy

Circular-dichroism spectroscopy experiments were carried out as described in Greenfield (52). The circular-dichroism spectra were measured on a JASCO apparatus in a 1 mm optical path cuvette for a wavelength range of 185–260 nm. Since Tris buffer gives a higher background signal in CD measurements, protein solution (wild-type and mutants) was prepared in 10 mM potassium phosphate pH 8 and 100 mM potassium chloride. The McrB mutants were not stable and precipitated during the thawing process, thus, before the start of the experiment, protein concentrations were re-estimated with Bradford reagent using BSA as a standard. Protein samples of 1.7 μM concentration were centrifuged at 21 000 g for 15 min to remove any precipitation immediately before collecting the CD spectra.

## RESULTS

### Oligomeric states of McrB in presence of GTP

**Figure 1.**
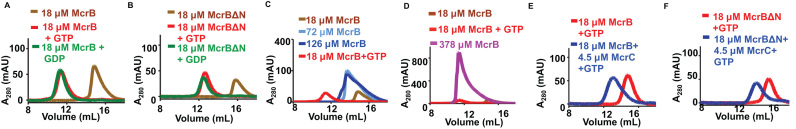
SEC studies on McrB and McrBΔN: (**A**) SEC elution profile of McrB using 24 ml Superdex 200 10/300 GL column. In the absence of GTP, the protein elutes at 14.85 ml while in the presence of GTP or GDP, the protein elution peak shifts to 11.44 ml. (**B**) SEC elution profile of McrBΔN using 24 ml Superdex200 10/300 GL column showing an elution peak shift from 15.8 ml (in the absence of GTP) to 12.64 ml (in the presence of GTP or GDP). (**C**) Gel filtration profile of different concentrations of McrB in the absence of GTP and comparison with McrB in the presence of GTP (using 24 ml Superdex 200 10/300 GL), showing different oligomeric states of the protein with increasing concentration of McrB even in the absence of GTP. (**D**) Gel filtration profile of 378 μM McrB in the absence of GTP (using 24 ml Superdex 200 10/300 GL) indicating the formation of a higher-order oligomer. (**E**) Gel filtration profile of McrB with and without McrC in the presence of GTP (using 24 ml Superose 6 10/300 GL column). (**F**) Gel filtration profile of McrBΔN with and without McrC in the presence of GTP (using 24 ml Superose 6 10/300 GL column). The study shows that in presence of McrC, the oligomeric McrB shifts from 14.8 to 13 ml and the oligomeric McrBΔN peak shifts from 15.7 to 14.2 ml.

Full length McrB and McrBΔN (amino acid 162–460) were purified by affinity and ion-exchange chromatography to ∼95% homogeneity (Supplementary Figure S4). An additional SEC column was used to purify the samples in presence of GTP. The bound nucleotide was removed by washing the eluted protein with buffer without nucleotide (see Materials and Method). The removal of nucleotide was confirmed by measuring absorbance of sample at 260 nm. Further, SEC studies with purified protein, carried out in absence of nucleotide, showed monomeric state of McrB or McrBΔN (Figure 1A, B). In presence of GTP or GDP, McrB and McrBΔN eluted as a high molecular mass oligomer (Figure 1A, B). This further confirmed that the washing of protein with buffer containing no nucleotide removed the bound nucleotide. SEC studies in presence of nucleotide indicated an apparent molecular mass (calculated using molecular marker standard shown in Supplementary Figure S1A, B) of ∼380 kDa for McrB, which is more consistent with an oligomeric form containing seven subunits (theoretical molecular mass = 372 kDa) than a hexamer (theoretical molecular mass = 325 kDa). However, the apparent molecular mass of McrBΔN was ∼210 kDa, which is more consistent with a six subunit-oligomer (theoretical molecular mass = 214 kDa) than a seven subunit-oligomer (theoretical molecular mass = 249 kDa).

In the absence of GTP or GDP and at a protein concentration of 18 μM, both McrB and McrBΔN eluted as a single peak with an apparent molecular mass of 64 kDa (theoretical Molecular weight = 54.2 kDa) and 39 kDa (theoretical Molecular weight = 35.6 kDa), respectively (Figure 1A, B), which are close to the values expected for their monomeric forms. However, McrB showed a concentration dependent oligomerisation even in the absence of GTP. At a protein concentration of 72 μM and 126 μM, McrB eluted as an inhomogeneous mixture of different molecular weight species, composed possibly of monomers, dimers, trimers etc (Figure 1C, D). At a much higher concentration (378 μM), McrB eluted as a higher order oligomer with an apparent molecular mass equivalent to the McrB-GTP oligomer (Figure 1D). McrB also formed a higher order complex with McrC in the presence of GTP. The McrBC complex had a molecular mass much larger than the McrB-GTP oligomer as observed by SEC using a 24 ml Superose6 10/300 GL column (Figure 1E). A similar complex was formed between McrBΔN-McrC-GTP (Figure 1F), consistent with the existing model of an assembly of McrBC complex made of two rings of McrB and two subunits of McrC (11). McrC on its own did not form higher ordered oligomers in presence of GTP, nor did a mix of McrB and McrC in absence of GTP (Supplementary Figure S1C).

As the shape of the macromolecules affect their mobility during SEC, the molecular weights determined based on molecular weight standards are not necessarily accurate. This possibly explains the disparity in the mass of oligomeric state of McrB and McrBΔN derived by SEC. In order to obtain absolute molecular weight of the protein oligomers, we studied them using SEC-coupled multi-angle light scattering (SEC-MALS). The technique combines the ability of SEC to fractionate material into different sized particles with light scattering technique and theory (53–55). Collecting the multi-angle light scattering allows one to determine the radius of gyration from the angular dependence of scattered intensity. When particles are too small to generate detectable angular variation, the additional angles see the same intensity and increase the statistics of the isotropic point scatter.

**Figure 2.**
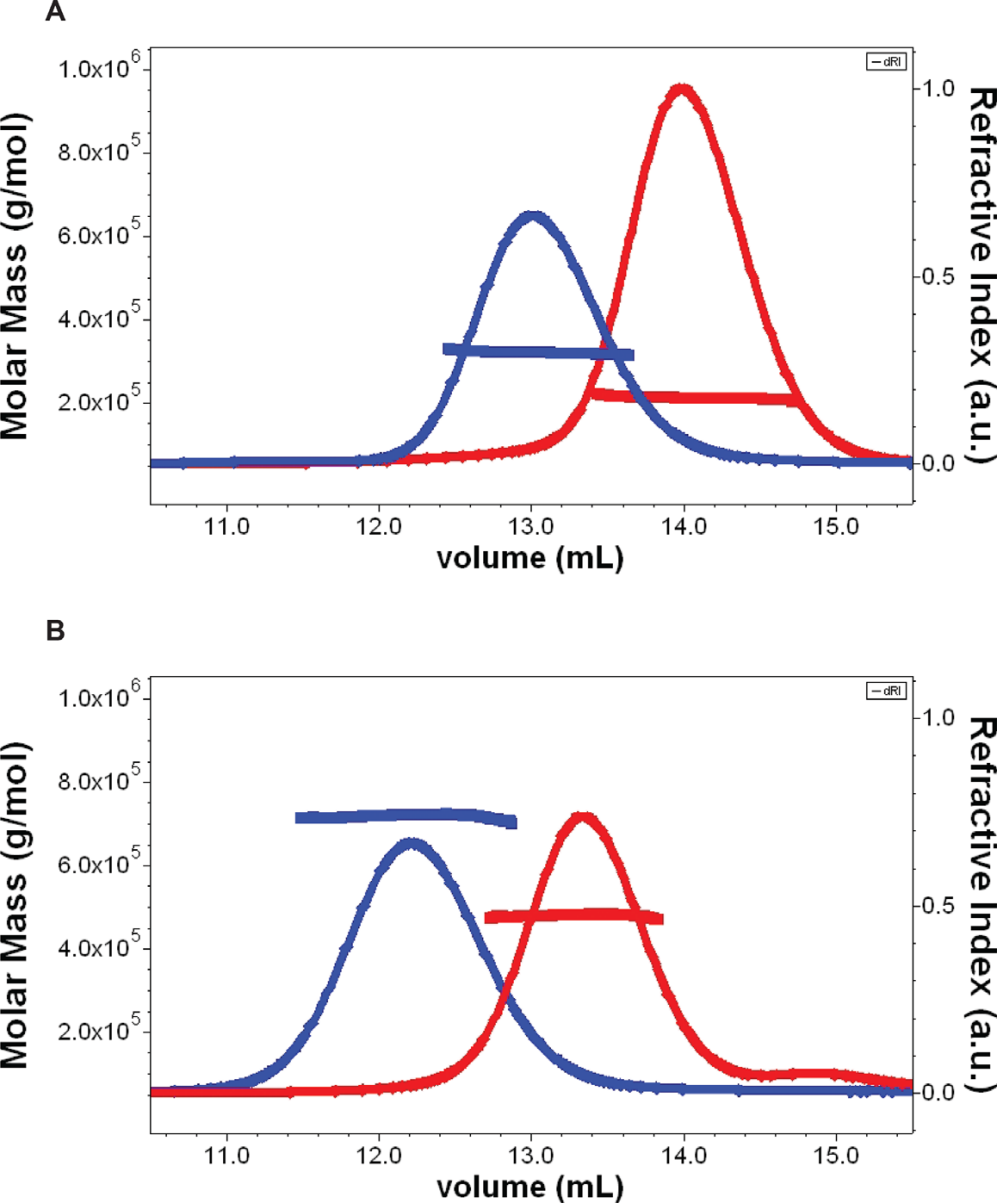
SEC-MALS chromatogram of McrB and McrBC: The chromatogram shows the refractive index signal with the derived molar masses indicated by the thicker horizontal lines. (**A**) McrB (blue) and McrBΔN (red) loaded at 0.6 and 0.8 mg/ml (protein concentration on column is about >1/10th of loaded concentration) displayed single highly monodisperse peaks with average mass over the indicated regions 320 kDa and 211 kDa, respectively. The R_h_ evaluated from DLS data over the same regions was 6.1 ± 0.2 and 5 ± 0.2 nm, respectively. (B) McrBC (blue) and McrBΔNC (red) run at 0.6 mg/ml displayed single highly monodisperse peaks with average mass over the indicated regions 720 and 486 kDa respectively. The R_h_ evaluated from DLS data over the same regions was 8.7 ± 0.2 and 6.7 ± 0.2 nm, respectively.

The SEC-MALS measurements clearly showed that in presence of nucleotide, both McrB and McrBΔN form a monodisperse peak (Figure 2A). The observed masses from SEC-MALS match relatively well with the calculated mass from the amino acid sequence for a hexameric McrB and McrBΔN (Figure 2A, Table 1). The complex of McrBC and McrBΔNC (McrBΔN without His6 tag) also showed monodisperse peaks and the molecular mass corresponds to the mass of a tetradecamer of 12 subunits of McrB plus 2 subunits of McrC. This suggested that McrBC is likely to be a heteromeric tetradecamer of two McrB hexamers and two McrC monomers (Figure 2B, Table 1). We did not observe self-association of McrB hexamers into higher oligomers in absence of McrC (Figure 2A), as was reported earlier (11). We also ran the samples at 1/10th of the concentrations shown in the figure (i.e. ∼0.05 mg/ml loaded and thus 0.005 mg/ml or less on the column) and the results were identical (data not shown).

**Table 1.** Comparison of calculated mass and mass from SEC-MALS measurements

	Oligomer	Mass from amino acid sequence	Mass from SEC-MALS
McrB	Hexamer	325 kDa	320 kDa
McrBΔN	Hexamer	214 kDa	211 kDa
McrBC	Tetradecamer (12 McrB +2McrC)	734 kDa	720 kDa
McrBΔNC	Tetradecamer (12 McrB +2McrC)	500 kDa	486 kDa

### SAXS analyses of the nucleotide-dependent oligomers

**Figure 3.**
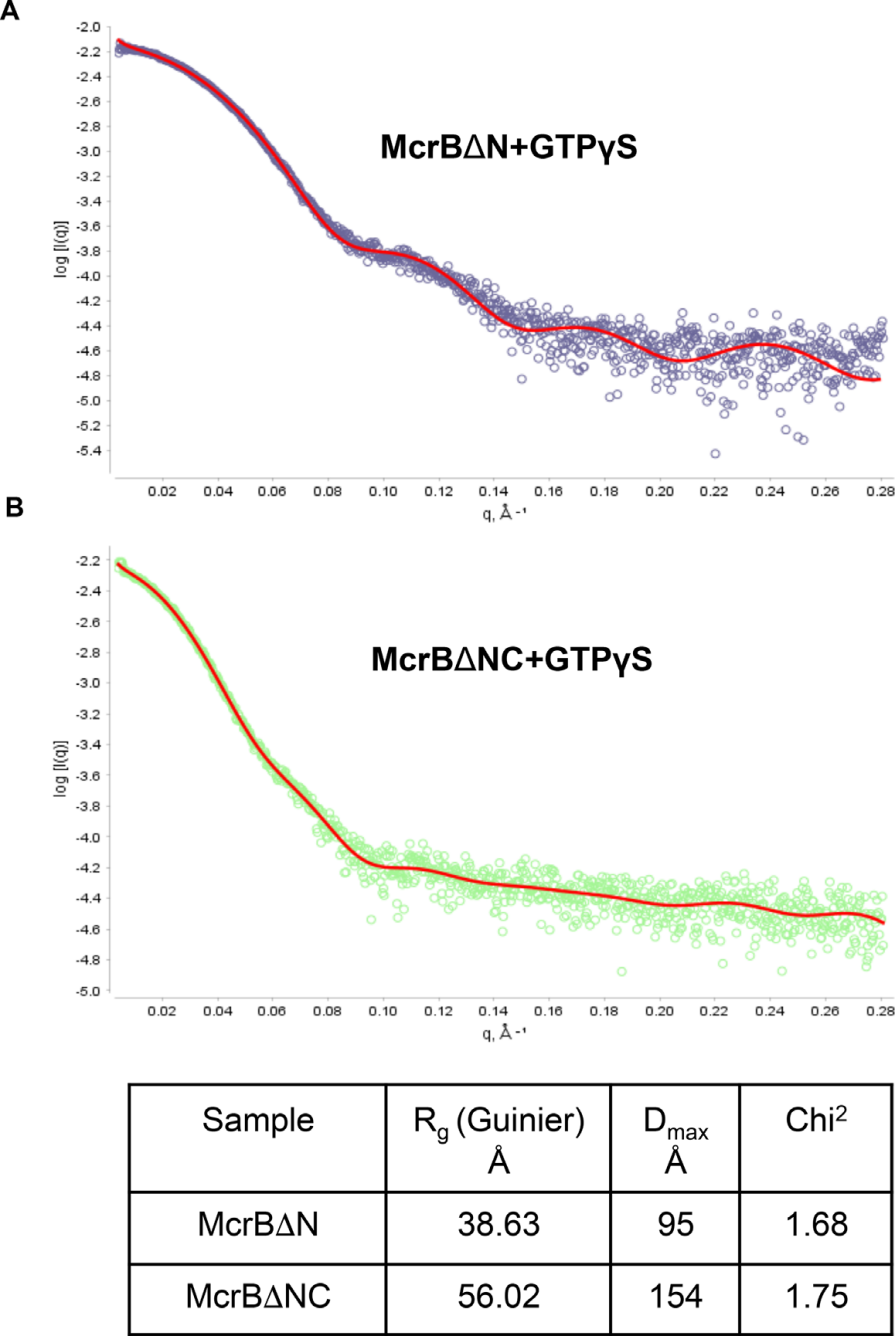
SAXS scatter plot of McrBΔN and McrBΔNC: The scatter plot of the processed SAXS data obtained for (A) McrBΔN and (B) McrBΔNC, analysed using SCATTER.

We also measured the molecular mass of the nucleotide-dependent oligomers using small angle X-ray scattering (SAXS) (39). Good quality data was collected for McrBΔN and McrBΔNC in presence of the non-hydrolysable analogue GTPγS. Aggregation prevented us from getting good data for McrB and McrBC. *R*_g_ was measured using SCATTER (Figure 3). SAXSMoW2 (54; http://saxs.ifsc.usp.br/) was used for obtaining the molecular mass using an integration range restricted to q*R*_g_ <1.3 and *q*_max_ limited to *I*(0)/*I*(*q*_m_) = 10^2.25^. The program can calculate molecular mass using a single SAXS curve measured on a relative scale with less than 10% error (56). The SAXS measurements carried out in presence of GTPγS gave a molecular mass of 197.5 kDa for McrBΔN and 476.4 kDa for McrBΔNC (Table 2). These values are close to that obtained using SEC-MALS and consistent with McrBΔN being a hexamer and McrBΔNC being a tetradecamer (12 subunits of McrB plus 2 subunits of McrC) in presence of the nucleotide.

**Table 2.** Molecular mass from SAXS analysis

Sample	Guinier limits (Å^−1^)	*I*_0_ (A.U.)	*R*_g_ (Å)	q*R_g_	Molecular mass (kDa)	% difference from calculated molecular mass
McrBΔN + GTPγS	0.005–0.032	0.007	39.90	1.279	197.5	7.7
McrBΔNC + GTPγS	0.006–0.022	0.006	58.63	1.299	476.4	4.7

*McrBΔN does not have His-tag.

### CryoEM 2D class averaged images of hexameric McrB

**Figure 4.**
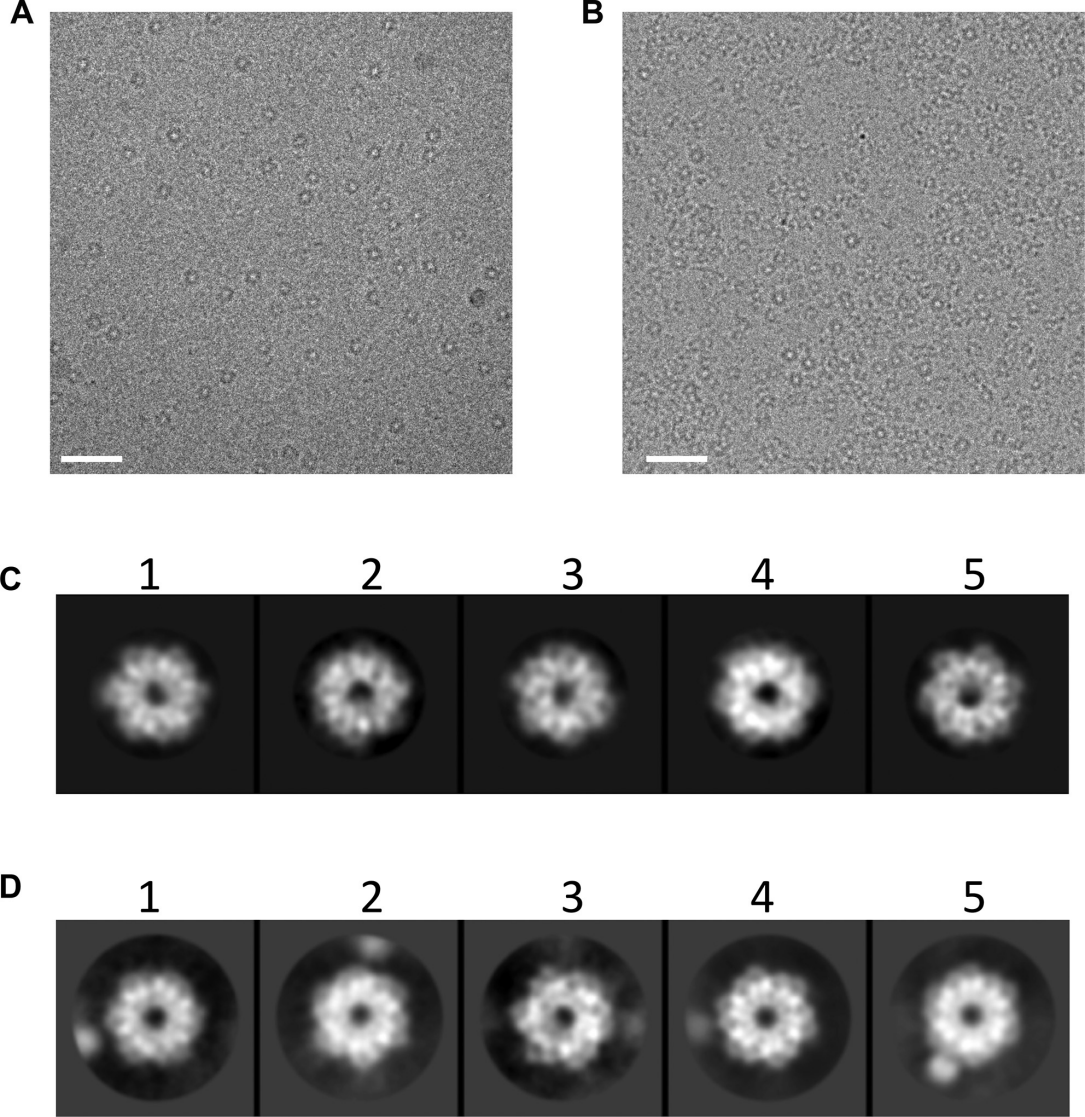
CryoEM images of McrB and McrBΔN in presence of GDPNP: (**A**) A small area of micrographs of McrBΔN, and (**B**) McrB showing round particles on ice. The images were captured with Falcon II CMOS detector. The scale bar is 400 Å. (**C**) Reference-free 2D class averages of McrBΔN, and (**D**) McrB. The box size is 160 pixels. McrBΔN was sampled at 1.33 Å/pixels and McrB at 1.35 Å/pixels. Approximate diameter of the particles is ∼95 Å and the central hole measures ∼25 Å. In McrB with the N-terminus, additional density beyond the ring is visible and this could be the N-terminus of the protein indicating flexibility in absence of DNA.

Having found that McrB/McrBΔN formed a homogenous hexameric oligomer in presence of GTP, we pursued structural characterisation of the complex using CryoEM. Imaging of McrB or McrBΔN after the addition of GDPNP showed round particles (Figure 4A, B). With present freezing conditions the particles showed preferred orientation predominantly showing the ‘top’/’bottom’ faces of the disc/toroid. Due to the use of a direct electron detector and higher defocus, protein hexamers could be occasionally visualised in raw images (Figure 4A, B). Reference-free two-dimensional (2D) class averages showed clearly the hexameric nature of both McrB and McrBΔN oligomers (Figure 4C, D). The hexamers appeared ring-shaped having a central pore. The outer diameter of the ring was ∼95 Å and the central pore diameter was found to be <25 Å. A number of hexameric AAA+ proteins have been found to form a spiral/lockwasher structure (18,21,57–59). As the top/bottom view of these structures would also resemble a ring, and since we do not have side views of McrB oligomer, we cannot conclude the shape of the oligomer from the available data.

The analyses of the rotational power spectrum of the class averages of McrBΔN using the program rfiltim showed a clear peak at six-fold (Supplementary Figure S5). Perhaps due to the additional density in McrB, the rotational power spectrum in a couple of classes of McrB had no clear peak at six-fold (Supplementary Figure S6). The density appeared fuzzy for these classes indicating heterogeneity. A similar observation was made previously from the negatively stained EM images of McrB (11). This possibly is indicative of the movement of the N-terminal DNA-binding domain, which is a structurally independent fold that can be expressed in isolation (12), and may be flexible when in fusion with the AAA+ domain.

### GTPase and endonuclease activity of McrBC

**Figure 5.**
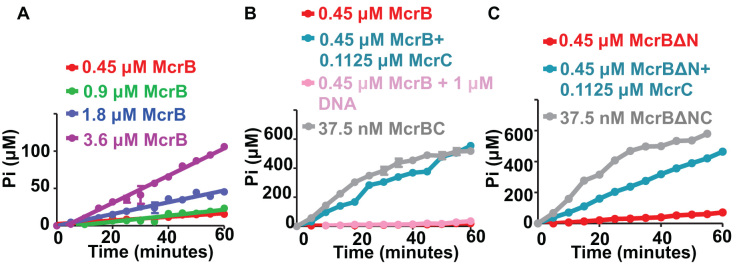
GTP hydrolysis by McrB and McrBC. (**A**) Time dependent GTPase activity of McrB at different concentrations. (**B**) Comparison of GTPase activity of McrB alone, McrB in presence of McrC, McrB in presence of 1 μM specific DNA and McrBC complex (assembled and purified using SEC; Mol wt 734 kDa). (**C**) Comparison of GTPase activity of McrBΔN alone, McrBΔN in presence of McrC and McrBΔNC complex (assembled and purified using SEC; Mol wt 500 kDa).

As the molecular mass of the oligomers of McrB, McrBΔN and McrBC were different from those reported earlier, we sought to find if the purified proteins were active. For this, we analysed the GTPase activity of the enzyme. A time-dependent release of phosphate ions upon hydrolysis of GTP was measured at three different concentrations of McrB and McrBΔN using a steady-state fluorimeter and a malachite green assay. The three concentrations were chosen from a McrB and McrBC concentration-dependent phosphate-release assay (Supplementary Figure S2B, C). We noticed only a weak GTPase activity for both McrB (Figure 5A) and McrBΔN (Supplementary Figure S2D). There was no significant change in the GTPase activity of McrB on addition of specific DNA (Figure 5B) or non-specific DNA (Supplementary Figure S2E). However, on addition of McrC to McrB or McrBΔN (molar ratio of 4:1 of McrB:McrC or McrBΔN: McrC), we noticed a significant increase in the release of phosphate (Figure 5B, C), as reported previously. 4:1 molar ratio was chosen so as to be consistent with previous studies (11,17,59).

**Figure 6.**
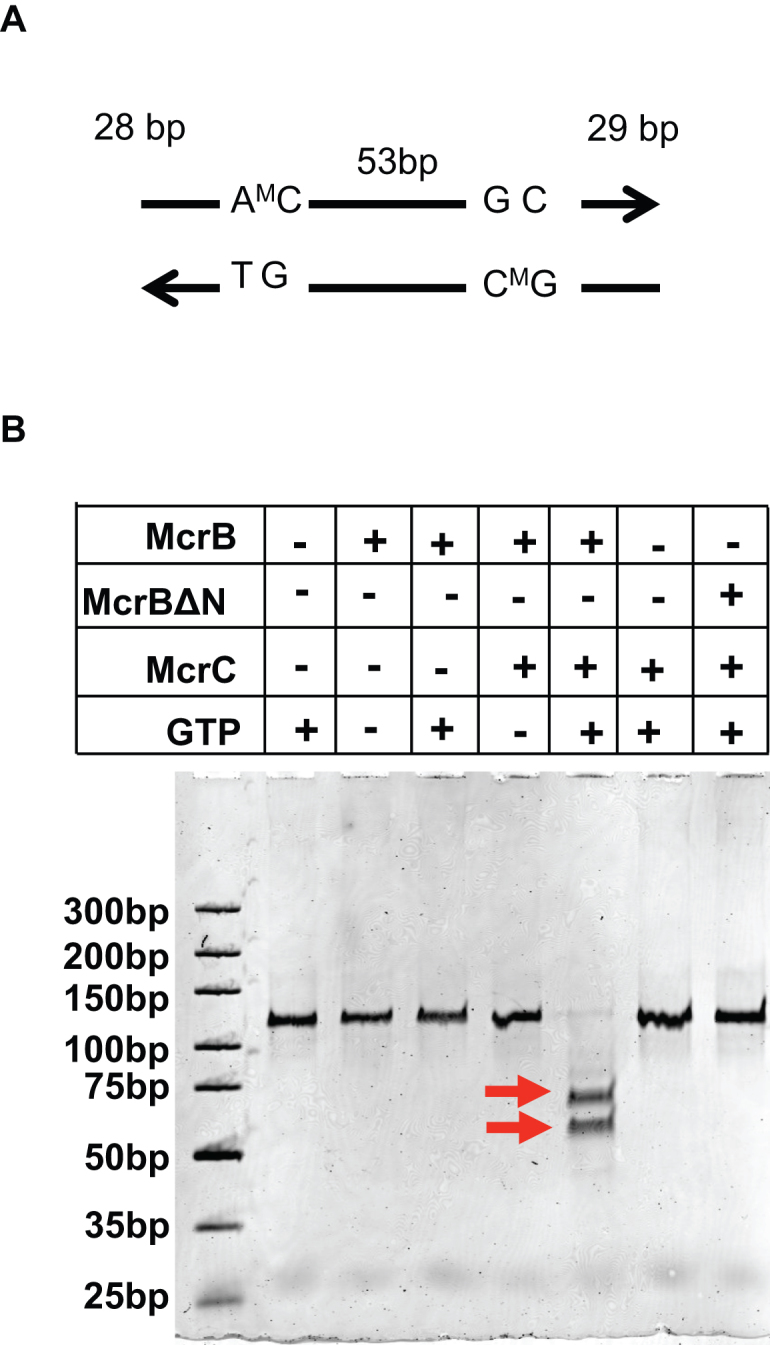
DNA cleavage assay. (**A**) Schematic representation of 114 bp substrate DNA used for DNA cleavage assay. (**B**) Native 10% (w/v) PAGE gel showing DNA cleavage activity of 900 nM McrB in the presence of 225 nM McrC, 75 nM substrate DNA and 1 mM GTP. The cleaved product is indicated by arrows.

We next tested the nucleolytic activity of the enzyme. A 114 bp DNA substrate containing two methylated target sites separated by 53 bp was used (Figure 6A). In presence of GTP, this substrate was readily cleaved by a mixture of McrB and McrC (4:1 molar ratio). Neither McrB alone, nor McrBΔN+McrC could cleave specific DNA in presence of GTP (Figure 6B). The McrBC complex purified by SEC retained its ability to cleave the substrate (Supplementary Figure S7). As expected, nucleolytic cleavage by McrBC yielded two products corresponding to a dsDNA break close to just one of the two target sites.

### Effect of Walker B mutants on McrB oligomerisation

In AAA+ proteins it is usually seen that mutation of residues interacting with the nucleotide affects nucleotide-dependent oligomerisation (60). Accordingly, mutation of the conserved Walker A lysine, which is involved in nucleotide binding, hinders nucleotide-dependent oligomerisation (60). On the other hand, mutation of the Walker B aspartate and glutamate residues is known to affect nucleotide hydrolysis but not its binding and protein oligomerisation (60). However, in case of McrB, a previous study reported that mutation of the Walker B residues prevented nucleotide binding (9). Consequently, we wondered if these mutants also affected the hexamerisation of McrB. To address this, we generated the McrB Walker B mutants McrB^D279A^, McrB^D279N^, McrB^E280A^ and McrB^E280Q^ and studied their oligomerisation properties.

**Figure 7.**
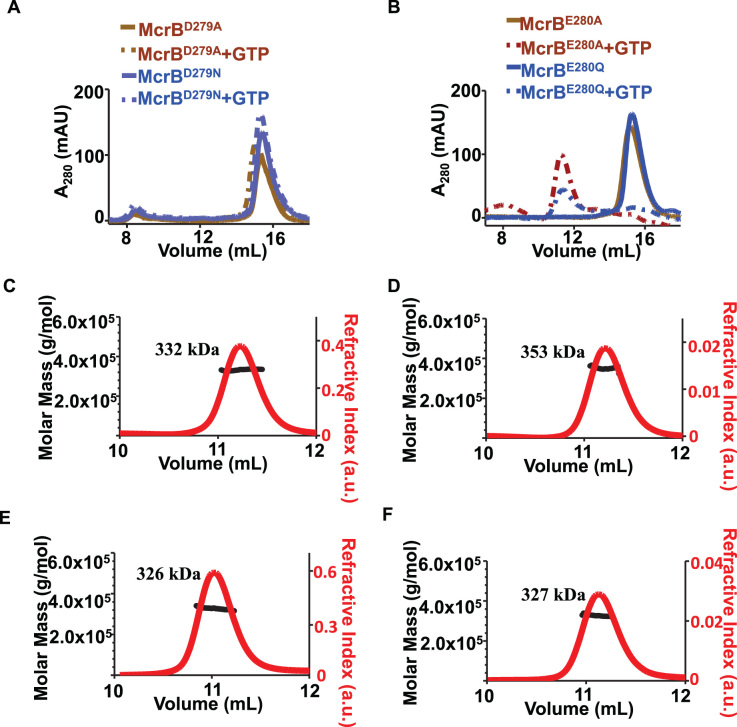
Affect of mutation of Walker B residues on McrB oligomerisation. (**A**) Gel filtration profile (using 24 ml Superdex200 10/300 GL), both in presence and absence of GTP, of 18 μM McrB^D279A^ and 18 μM McrB^D279N^. (**B**) Gel filtration profile (using 24 ml Superdex200 10/300 GL), both in presence and absence of GTP, 18 μM McrB^E280A^, 18 μM McrB^E280Q^. Chromatogram showing the refractive index signal with the derived molar masses indicated by the thicker horizontal lines for (**C**) McrB^E280A^ in presence of GTP, (**D**) McrB^E280A^ in presence of GDP, (**E**) McrB^E280Q^, in presence of GTP and (**F**) McrB^E280Q^ in presence of GDP. Protein concentration used in all the runs was 18 μM. The elution peaks displayed highly monodisperse population with average mass indicated over the region in each panel.

**Figure 8.**
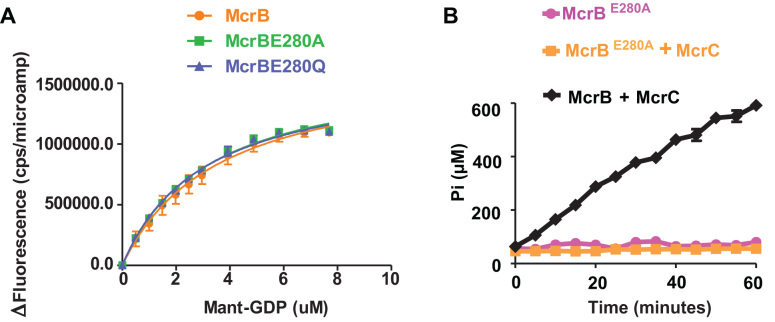
Effect of mutation of Walker B glutamate on GTP binding and hydrolysis. (**A**) Mant-GDP binding by McrB and its mutants. Change in Mant-GDP fluorescence, induced as a function of binding of 0.5 μM protein to Mant-GDP, is shown at different concentrations of the Mant-GDP. (**B**) Time dependent GTPase activity of McrB^E280A^ with and without McrC.

The far-UV CD spectra of the mutants suggested proper folding (Supplementary Figure S8). On analysing the oligomeric state of the mutants using SEC, we found that both McrB^D279A^ and McrB^D279N^ failed to oligomerise in presence of GTP and eluted at an elution volume corresponding to monomeric species (Figure 7A). This observation is consistent with the previous report (9) and our observation (Supplementary Figure S9) that McrB^D279A^ and McrB^D279Q^ do not bind nucleotide. However, McrB^E280A^ and McrB^E280Q^ eluted at an elution volume corresponding to hexameric species (Figure 7B). Further analysis using SEC-MALS showed that both McrB^E280A^ and McrB^E280Q^ had a mass comparable to wild type McrB hexamer in presence of GTP or GDP (Figure 7C–F). Analysis of nucleotide binding using Mant-GDP revealed that both McrB^E280A^ and McrB^E280Q^ bound to the nucleotide as well as the wild type (Figure 8A). But the mutants were deficient in nucleotide hydrolysis (Figure 8B).

### GTP hydrolysis follows oligomerisation of McrBC

**Figure 9.**
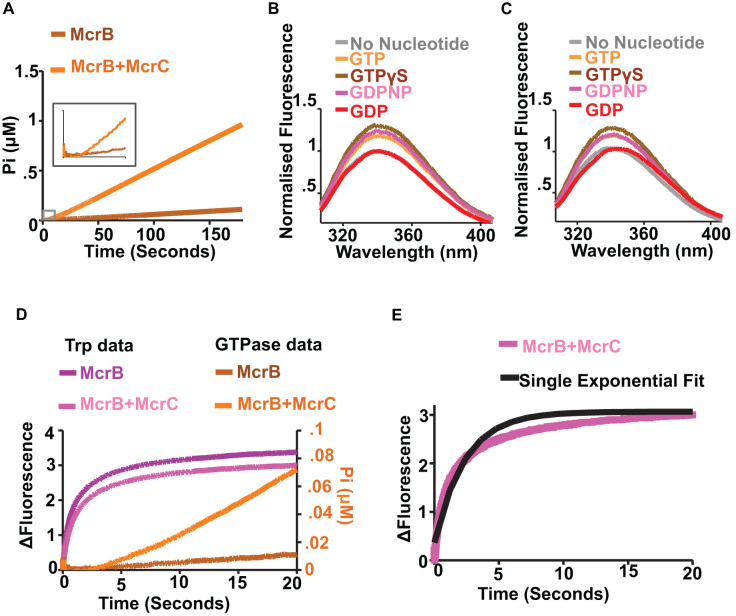
Relation between GTP hydrolysis and oligomerisation. (**A**) Observation of GTPase activity with 0.5 μM McrB and 0.5 μM McrB+0.125 μM McrC when the protein was mixed with 1 mM GTP. The inset shows the first 10 seconds of GTPase activity of both McrB and McrB+McrC, indicating a lag phase. (**B**) Steady state tryptophan fluorescence emission scan of 500 nM McrB in presence of different nucleotides. (**C**) Steady state tryptophan fluorescence emission scan of 500 nM McrB and 125 nM McrC in presence of different nucleotides. (**D**) Comparison of time trace of tryptophan fluorescence of 0.5 μM McrB and 0.5 μM McrB + 0.125 μM McrC (mixed with 1 mM GTP) with time trace of GTPase reaction carried out at similar protein and nucleotide concentrations. (**E**) Fitting of Trp fluorescence data of 0.5 μM McrB+0.125 μM McrC (mixed with 1 mM GTP) with single exponential model.

We next performed pre-steady state analysis of GTP hydrolysis by stopped-flow measurements of phosphate ion release. The GTPase activity was measured by mixing McrB+McrC (4:1 molar ratio) with GTP using the phosphate binding protein (PBP), which produces an increase in fluorescence upon rapid and tight binding to free phosphate (48,50). The use of rapid mixing to measure the GTPase activity has a distinct advantage over previous steady-state studies, which used techniques that required long measurement times (in order to stabilise the signal). Consistent with our previous observations, we observed stimulated GTPase activity of McrB in presence of McrC. Also, the profile for the rate of GTP hydrolysis showed a lag before steady state of GTP hydrolysis was reached (Figure 9A). The lag was observed irrespective of the presence of McrC (Figure 9A; inset). As nucleotide hydrolysis by AAA+ proteins require oligomerisation of the subunits in general, we wondered if the lag signified the time required for oligomerisation of McrB and McrBC.

To study nucleotide-dependent oligomerisation of McrBC, we investigated if intrinsic Trp fluorescence of the protein can be used as a signal. McrB has six tryptophans - three in the N-terminal DNA binding domain and three in the C-terminal AAA+ domain, while McrC has four tryptophans. Steady-state fluorescence was used to measure the changes in the emission profiles upon addition of GTP (Figure 9B). McrB showed ∼20% increased tryptophan fluorescence in the presence of GTP. A similar shift was observed in the presence of non-hydrolysable analogues of GTP, GDPNP and GTPγS, resulting in ∼20% and ∼30% increase in tryptophan fluorescence without any spectral shift, respectively (Figure 9B). In contrast, the presence of GDP did not increase tryptophan fluorescence (Figure 9B) despite promoting hexamerisation (Figure 1A).

Addition of McrC to McrB did not affect the fluorescence signal (Figure 9C). However, addition of nucleotide to McrB+McrC mix (4:1 molar ratio) showed enhancement of the signal (Figure 9C). The change in signal was ∼15% in the case of GTP and GDPNP, and ∼23% in case of GTPγS (Figure 9C). As in the case of McrB, fluorescence change was not observed on addition of GDP (Figure 9C) to the McrB + McrC mix. This suggested that there is a difference in the structural states of the oligomers formed in complex with GTP and GDP and/or that the γ phosphate modulated the environment of one or more tryptophans. For example, it has been shown that the three dimensional structure of certain AAA+ proteins, such as SV40 Large tumor antigen, in the ATP-bound state is different from that in the ADP-bound state (18). It is possible that a similar difference exists in the different nucleotide-bound states of McrB. As a consequence, fluorescence emission increased upon binding of nucleotide having a γ phosphate.

Identical experiments carried out at different times had wavelength of emission maxima varying from 337 to 341 nm in case of both McrB and McrB+McrC (data not shown) when nucleotide was not added. However, in presence of nucleotide (including GDP), the wavelength of emission maxima was always 341 or 342 nm. As change in fluorescence signal was noted in presence of either GTP or its non-hydrolysable analogues, we concluded that this change was a result of GTP binding and/or the nucleotide-dependent oligomerisation of the subunits.

We next monitored the change in Trp fluorescence intensity of McrB or a mix of McrB and McrC (McrB+McrC) over time after rapid-mixing with nucleotide. 1 μM McrB or 1 μM McrB + 0.5 μM McrC was mixed with 2 mM GTP in 1:1 ratio (final concentration were 0.5 μM McrB, 0.125 μM McrC (if present) and 1 mM GTP after mixing) using the stopped-flow apparatus and the corresponding change in fluorescence emission was measured (Figure 9D). The fluorescence profile from McrB and McrB+McrC were similar. We also measured phosphate release by McrB or McrB + McrC by mixing protein and GTP at identical concentrations. Plotting the GTPase and Trp fluorescence profile together revealed that the steady-state hydrolysis was reached only when maximum Trp fluorescence intensity changes were attained (Figure 9D). As the change in Trp fluorescence signal is due to GTP-binding and/or the subsequent oligomerisation, the lag observed in the GTPase profile represented one of these events.

If the Trp signal were due to the formation of a higher order species, then one would expect to observe a lag in the signal due to the multiple steps needed to form the oligomer. However, if the first step in the oligomerisation or the association of the nucleotide were to trigger the Trp signal, one would expect an exponential dependence without a lag. The tryptophan data does not clearly show a lag and cannot be fitted to a single exponential (Figure 9E). Since the change in the Trp signal could come from any combination of species (dimer, trimer, hexamer, etc.), this is hard to model. Thus, though the Trp change we observed might be a signal on pathway to oligomer formation, we cannot necessarily determine if it is the end point of the reaction. Due to the complexity of the signals, we did not further investigate the dependence on the concentration of nucleotides. Thus, the Trp response with GTP and its absence with GDP suggest that it is linked not only to the binding pocket, but also to higher order changes that we interpret as oligomerisation and hydrolysis activation.

**Figure 10.**
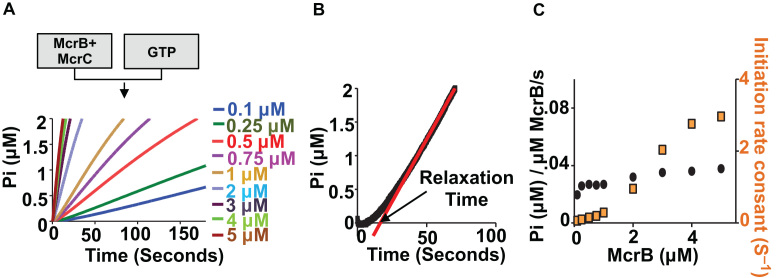
Relation between GTP hydrolysis and oligomerisation: (**A**) GTPase data for McrB+McrC collected at different concentrations of protein. McrB concentration is mentioned in the figure while corresponding McrC concentration was 1/4th of McrB concentration. Measurements were carried out with 1 mM GTP. (**B**) An example of data analysis showing the linear straight line fit for steady-state rate and intercept at X-axis for initiation time constant (reciprocal of relaxation time). (**C**) Graph showing effect of protein concentration on steady-state GTP hydrolysis rates and rate at which steady-state is attained (Initiation).

To explore if complete oligomerisation of McrBC preceded steady-state GTP hydrolysis, the GTPase rate was measured with increasing concentrations of McrB+McrC in the presence of saturating (1 mM) GTP (Figure 10A). In each case, the McrB:McrC molar ratio was maintained at 4:1. If complete oligomerisation preceded hydrolysis, one would expect that the steady-state phase would return the same microscopic rate (i.e. phosphates released per McrB) irrespective of the concentration of McrBC, while the lag phase, from a simple consideration of the law of mass action, would decrease with an increase in concentration. The steady-state rate and an apparent lag time were estimated by fitting the steady-state phases by straight line, where the gradient is the steady-state rate and the X-axis intercept is the apparent lag time (relaxation time). The reciprocal of the relaxation time gave an apparent initiation rate constant (Figure 10B). The relationship between steady-state rates (μM Pi/μM McrB/s) and concentration of McrB was approximately constant (Figure 10C). In contrast, the apparent initiation rate was dependent on protein concentration. This observation indicated that the lag in GTP hydrolysis resulted from a requirement for McrBC to oligomerise first.

## DISCUSSION

An integral feature of proteins containing AAA+ domain is their oligomerisation into functional units. We carried out studies on assembly of McrB oligomer and McrBC complex, toward understanding the mechanism of this restriction enzyme. In addition, studies with McrBΔN, a variant form of the protein lacking the N-terminal DNA-binding domain, were also carried out. SEC studies confirmed that monomeric McrB and McrBΔN assembled into a higher-order oligomeric structure in presence of GTP, GDP or GDPNP (Figure 1A, B), which could be disassembled by washing away the nucleotides. Similar observations have been made in case of other AAA+ proteins, such as SV40 large tumor antigen, ClpAP, ClpB and ClpX, where these proteins dissociate into monomers, dimers, trimers or tetramers when nucleotide is absent, but assemble into hexamers once nucleotide is added (61–64). McrB also showed a concentration-dependent oligomerisation in absence of nucleotide. At lower protein concentrations, McrB predominantly existed as monomers (Figure 1A). But at very high concentration of the protein, a mixture of intermediate-size and higher-order oligomers was observed (Figure 1D). Concentration-dependent, nucleotide-independent oligomerization has been reported in case of other AAA+ proteins like Rep68 and ClpB (28,64). Finally, a complex of McrBC was assembled and purified from a mix of 4:1 molar ratio of McrB and McrC, GTP.

Analysis by SEC-MALS showed clearly that in presence of nucleotide both McrB and McrBΔN existed as highly monodisperse hexamers in solution. In presence of GTP McrB and McrBΔN assembled into a hexamer, while McrBC and McrBΔNC assembled into an oligomer made of twelve subunits of McrB and two subunits of McrC. This was further substantiated using SAXS. The hexameric structure of McrB and McrBΔN was further confirmed by cryoEM and 2D class averages. In a previous study, a heptameric structure was deduced using SEC, negative stain EM and mass analysis by STEM (11). The accuracy of molecular mass deduced from the retention volume of molecules obtained by SEC analysis is greatly affected by their shape; the non-native staining artefacts and the quality of images obtained by EM can contribute to errors in characterisation of the oligomeric structure; the mass analysis by STEM for a molecule in the mass range of McrB and McrBC can have error of ∼10%, and is not sensitive enough to determine the number of subunits (65). However, the techniques of SEC-MALS, SAXS and cryoEM that maintain the native state of the oligomer, unequivocally demonstrate that both McrB and McrBΔN are hexamers in presence of GTP.

Using Trp fluorescence we could monitor the nucleotide-dependent oligomerisation of McrBC. Comparison of the trace of McrBC oligomerisation and GTPase reaction kinetics, revealed that the nucleotide hydrolysis achieved steady-state only on oligomerisation of McrBC. The absence of Trp signal in steady state experiment with GDP suggested that the change in Trp fluorescence was mostly due to γ-phosphate specific conformations. Since SEC studies clearly showed that the protein oligomerises in presence of GDP, it appears that the signal is probably specific for a GTPase active state attained upon GTP binding which subsequently leads to oligomerisation. The Trp signal change corresponded to the lag phase observed in GTPase study under similar mixing regime. This lag was found to be dependent on protein concentration in presence of saturating GTP. Taking into consideration these observations, we interpret that the lag observed in the GTPase profile is due to oligomerisation of McrBC, which precedes the steady state GTP hydrolysis. This conclusion is in accordance with the general understanding that AAA+ proteins are functional only as oligomers.

A unique feature of the oligomerisation of McrB is the importance of the Walker B aspartate. Unlike most other AAA+ proteins where mutation of the aspartate does not affect nucleotide-dependent oligomerisation but only hampers nucleotide hydrolysis, in case of McrB mutation of the aspartate to alanine or asparagine affected oligomerisation. This aspartate is expected to form coordination bond with the nucleotide-bound magnesium (66). The mutational studies suggest that in McrB the aspartate may also contribute to stabilising inter-subunit interaction. In contrast, we found that mutation of the Walker B glutamate, which is predicted to activate the catalytic water, to alanine or glutamine only affects nucleotide hydrolysis, without affecting nucleotide binding or oligomerisation. This result is in contrast with previous report showing loss of GTP binding upon mutation of Walker B glutamate (9) but in line with the putative role of Walker B glutamate as the catalytic base required for nucleotide hydrolysis (60,66).

**Figure 11.**
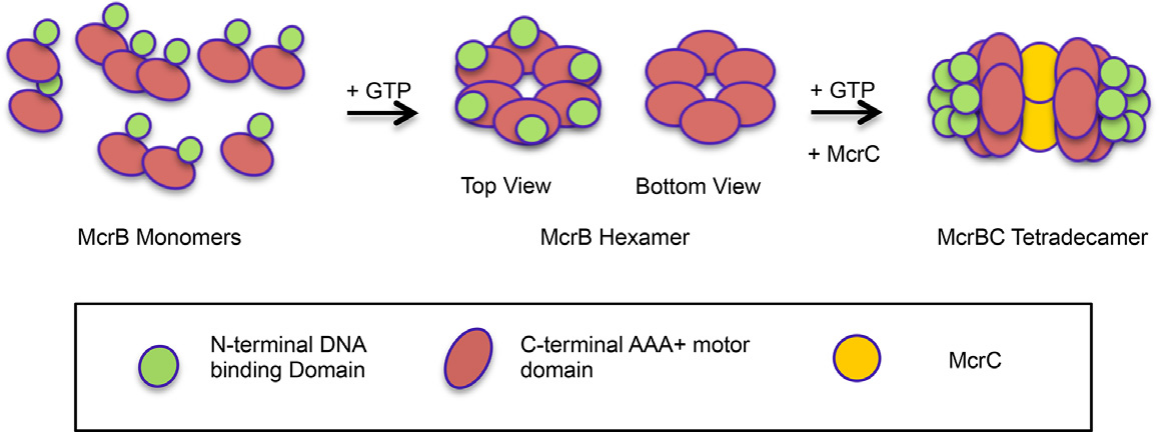
A model of the oligomeric assembly of McrB and McrBC complex.

In summary, we conclude that the functional unit of McrB is a hexamer and that the McrBC complex is made of the two McrB hexamers bound to two subunits of McrC. As McrC on its own was prone to precipitation, we could not find its oligomeric state. However, like most other endonucleases, McrC may exist as a dimer. The observation that both McrB and McrBΔN can complex with McrC, and that McrC may exists as a dimer leads us to propose that the McrBC complex is made of two rings of McrB sandwiching a dimer of McrC, with the N-terminal DNA binding domains pointing outward (Figure 11). It is tempting to speculate that the dimer of McrC would get activated upon collision of two assemblies of McrBC translocating DNA through the pore of the ring. The activated nuclease dimers of the McrBC bound to the target sequence would then nick the two strands of DNA resulting in a dsDNA break.

The oligomeric assembly of McrB reported here also provides insights into its interaction with DNA. The diameter of central pore of McrB hexameric ring observed in this study is large enough to accommodate dsDNA being threaded through the ring. But a closed ring shape of McrB imposes topological constraint in loading onto long, non-linear DNA which are readily cleaved by McrBC (17). It is possible that the hexameric ring of McrB could open up on complexation with McrC. Alternatively, the hexameric rings could disassemble into monomers and other oligomeric intermediates, which could then reassemble on the DNA substrate to form the functional oligomeric complex with McrC. To better understand this process and gain further mechanistic insights, we are pursuing biochemical, biophysical and structural studies of McrBC.

## Supplementary Material

Supplementary Data
