# Supplementary material for: Hexameric assembly of the AAA+ protein McrB is necessary for GTPase activity
Source: Nucleic Acids Res. 2018 Dec 6;47(2):868–82. doi: 10.1093/nar/gky1170 (PMC6344862; doi:10.1093/nar/gky1170)
Supplement: Supplementary Data [file gky1170_supplemental_files.pdf]

Supplementary Information

Hexameric assembly of the AAA+ protein McrB is necessary for GTPase activity

Neha Nirwan[1], Pratima Singh[1], Gyan Gourab Mishra[1], Christopher M. Johnson[2], Mark D. Szczelkun[3], Katsuaki Inoue[4], Kutti R. Vinothkumar[2!], Kayarat Saikrishnan[1*]

[1]*Division of Biology, Indian Institute of Science Education and Research, Pune, 411008, India.*

[2]*MRC Laboratory of Molecular Biology, Cambridge, CB2 0QH, UK.*

[3]*DNA-Protein Interactions Unit, School of Biochemistry, Medical Sciences Building, University of Bristol, Bristol BS8 1TD, UK*

[4]**Diamond Light Source, Harwell Science and Innovation Campus, Fermi Avenue, Didcot, OX11 0DE, UK.**

[*]*Corresponding author*

Phone: +91 2025908047
Fax: +91 2025908186

email: saikrishnan@iiserpune.ac.in

[!]*current address – National Centre for Biological Sciences-TIFR, GKVK Post, Bellary Road, Bangalore 560065.*

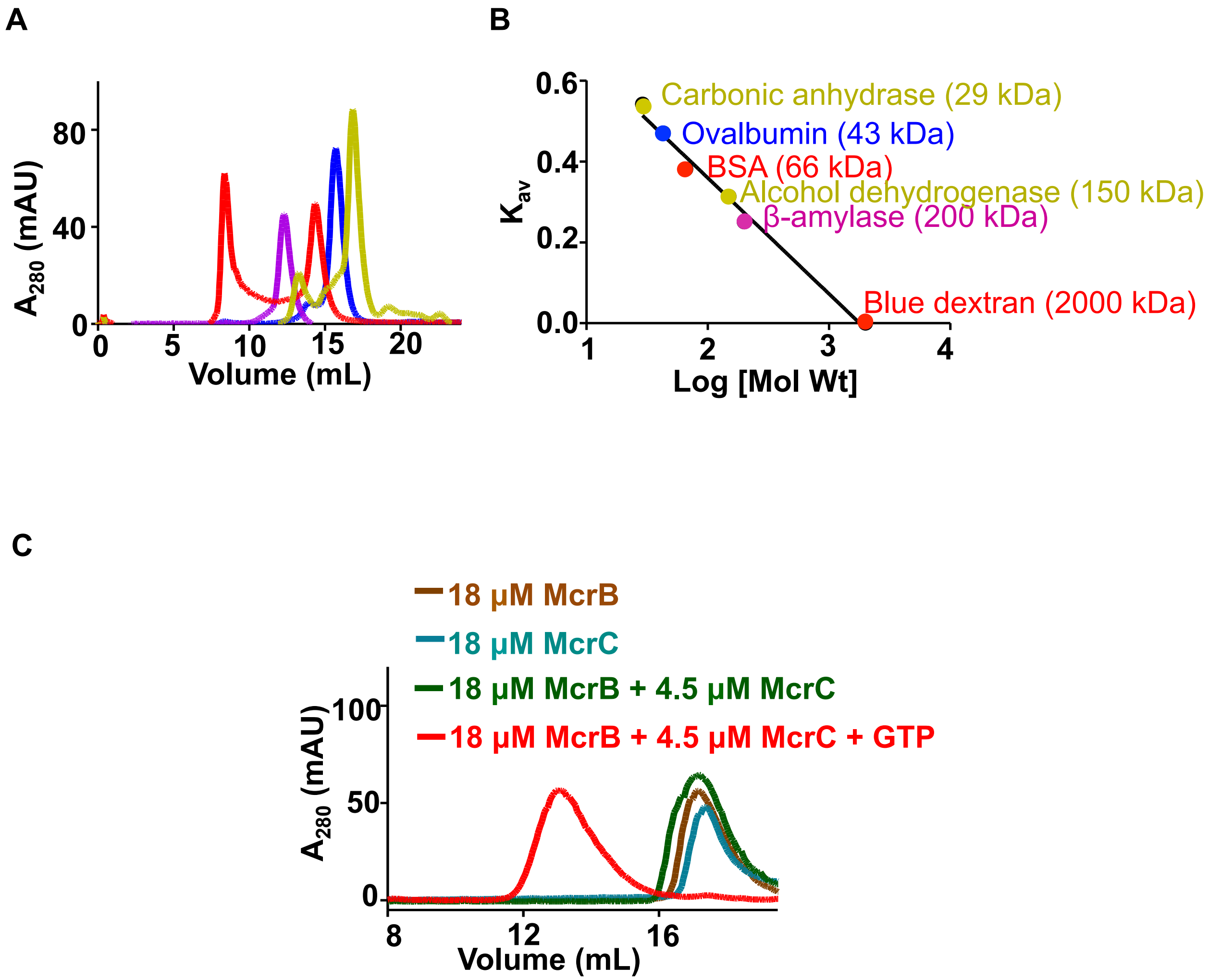

Supplementary Figure 1. A) Separation of different standard molecular weight markers on Superdex200 10/300 GL column run at 0.5 ml/min. B) Standard curve, obtained from standard molecular weight markers, used to predict the molecular weight of McrB and its oligomers. C) SEC analysis of McrC and McrB + McrC using a 24 ml Superose6 10/300 GL column . The injection volume in each case was 500 μL.

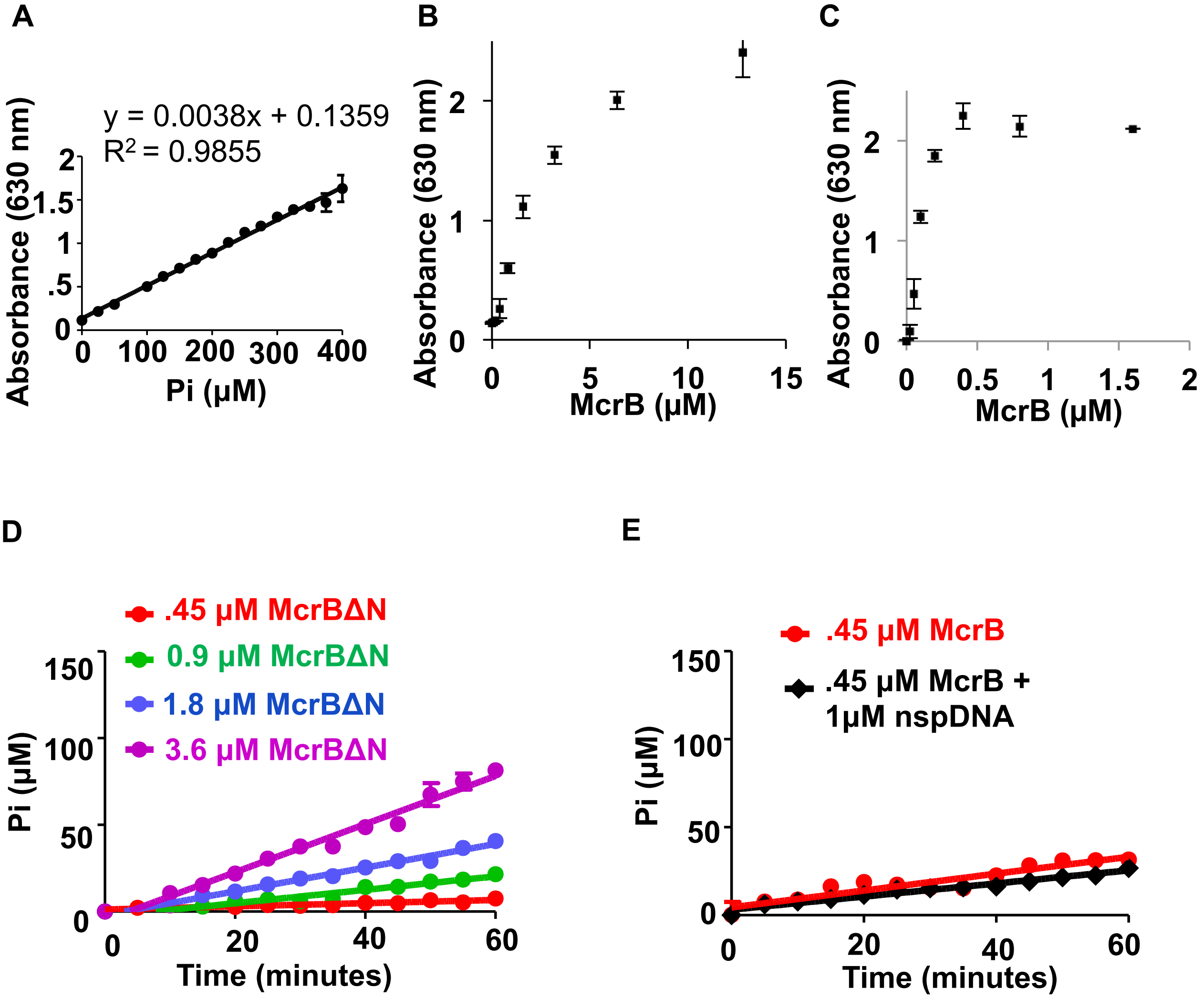

Supplementary Figure 2. A) The phosphate standard curve showing the absorbance at 630nm Vs increasing amount of Pi (NaH_2PO_4). B) Concentration dependent GTPase activity of McrB in presence of 1 mM GTP, C) Concentration dependent GTPase activity of McrB in presence of McrC (added at $1/4^{th}$ of McrB molar concentration at each data point) and 1 mM GTP, D) time dependent GTPase activity of McrBΔN at different concentrations, and E) comparison of time dependent GTPase activity of 450nM McrB alone and in presence of 1 μM non-specific DNA (nspDNA)

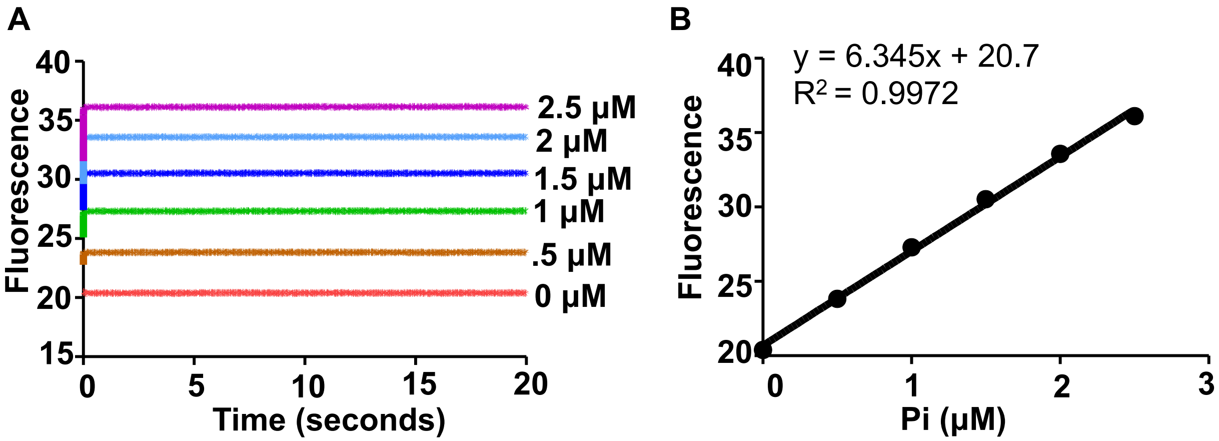

Supplementary Figure 3. A) Fluorescence measurement of 6 μM (final concentration) PBP-MDCC at varying concentrations of phosphate (Pi) over time. B) The phosphate (Pi) standard curve obtained from data shown in panel A. The different concentrations of Pi shown in figure are final Pi concentrations after mixing PBP-MDCC and Pi in 1:1 ratio.

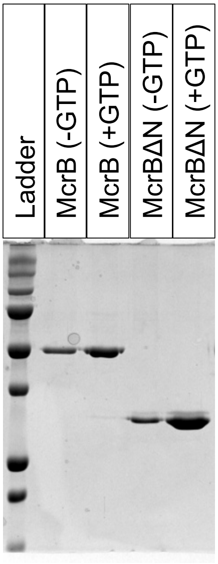

Supplementary Figure 4. SDS PAGE gel showing purified McrB and McrBΔN proteins both in presence and absence of GTP.

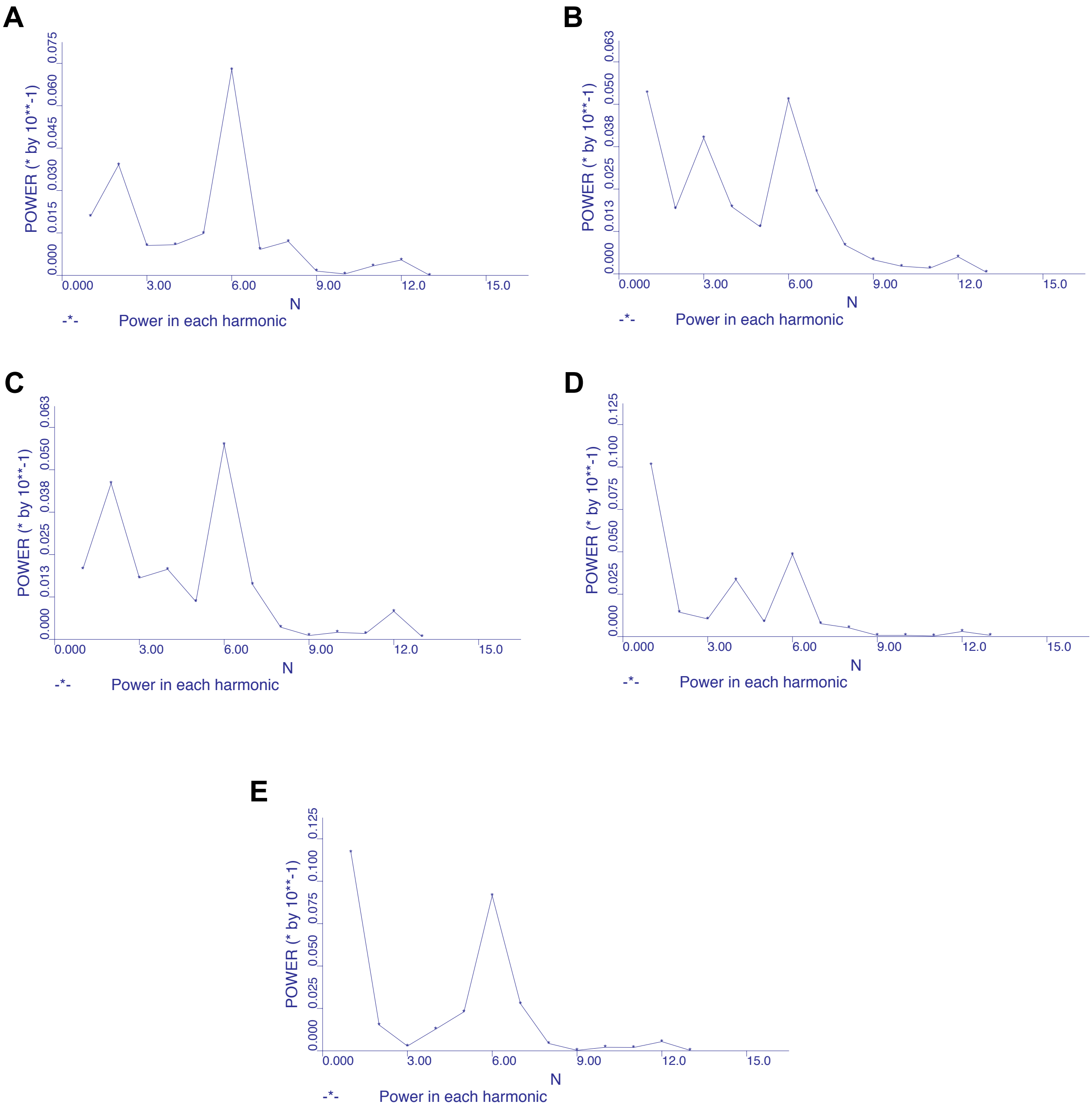

Supplementary Figure 5. Representative rotational power spectrum for class averages in A) Figure 4C-1, B) Figure 4C-2, C) Figure 4C-3, D) Figure 4C-4, E) Figure 4C-5. The Fourier transforms of the 2D images are filtered with a specified symmetry and the peaks reflect the power in each harmonic. In all the above panels, a peak at 6 is the most prominent. Our attempts at using other symmetry (in this case 5 or 7) showed no corresponding peaks.

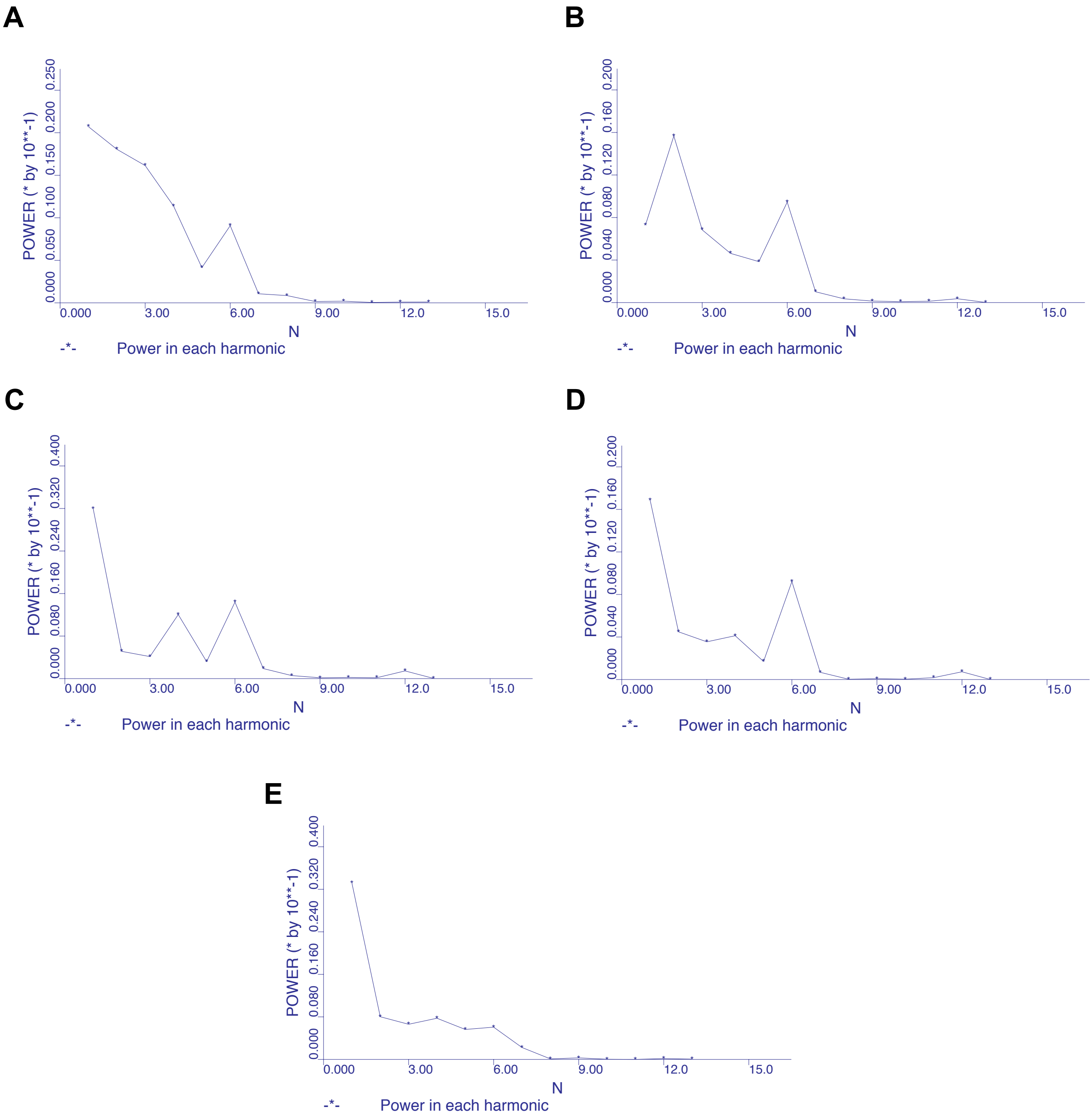

Supplementary Figure 6. Representative rotational power spectrum for class averages in A) Figure 4D-1, B) Figure 4D-2, C) Figure 4D-3, D) Figure 4D-4, E) Figure 4D-5.

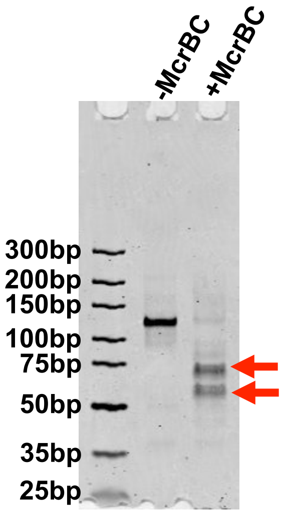

Supplementary Figure 7. Native 10% (w/v) PAGE gel showing DNA cleavage activity of 75 nM McrBC complex (assembled and purified using size exclusion chromatography) in presence of 75 nM of substrate DNA and 1 mM GTP. The cleaved product is indicated by red arrows.

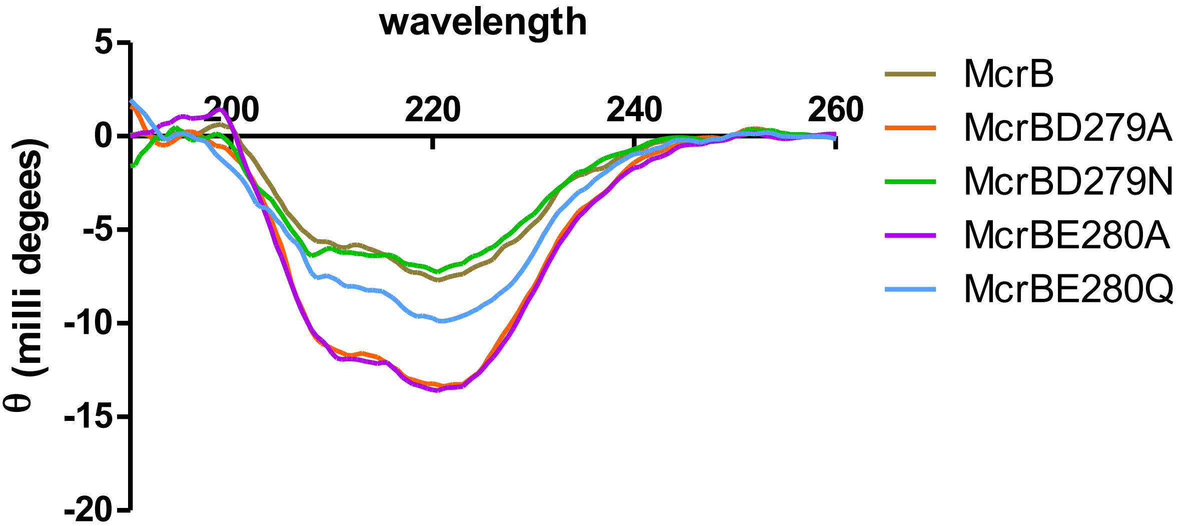

Supplementary Figure 8. Circular Dichroism spectroscopy of McrB and its mutants. The circular dichroism spectra of McrB and its Walker B mutants at 25ºC. An initial concentration of 1.7 μM protein was used for the experiments. The variation in the amplitudes could be a result of change in concentration of the proteins from the initial estimate due to protein precipitation. Prior to the measurement, all the samples were centrifuged to remove aggregates/ precipitates.

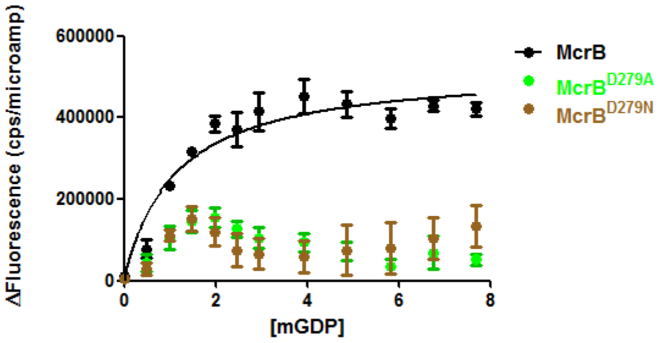

Supplementary Figure 9. Mant-GDP binding by McrB^{D279A} and McrB^{D279Q} mutants.

Supplementary Table 1. **List of primers used to generate McrB mutants.**

**Clone**	**Forward primer Sequence (5' -> 3')**	**Reverse primer Sequence (5' -> 3')**
McrB^{D279A}	GCCAGAGAAAAAGTATATTTTTATTATAGCTGAAATCAATCGTGCC	GATGATGGGATCCCGATGAGTCCCC
McrB^{D279N}	GCCAGAGAAAAAGTATATTTTTATTATAAATGAAATCAATCGTGCC	GATGATGGGATCCCGATGAGTCCCC
McrB^{E280A}	GCCAGAGAAAAAGTATATTTTTATTATAGATGCAATCAATCGTGCC	GATGATGGGATCCCGATGAGTCCCC
McrB^{E280Q}	GCCAGAGAAAAAGTATATTTTTATTATAGATCAAATCAATCGTGCC	GATGATGGGATCCCGATGAGTCCCC
